# UK Medical Cannabis Registry: A Clinical Outcomes Analysis for Complex Regional Pain Syndrome

**DOI:** 10.1002/brb3.70823

**Published:** 2025-09-02

**Authors:** Lilia Evans, Simon Erridge, Madhur Varadpande, Arushika Aggarwal, Isaac Cowley, Evonne Clarke, Katy McLachlan, Ross Coomber, James J. Rucker, Michael Platt, Mikael H. Sodergren

**Affiliations:** ^1^ Medical Cannabis Research Group Imperial College London London UK; ^2^ Curaleaf Clinic London UK; ^3^ St. George's Hospital NHS Trust London UK; ^4^ Department of Psychological Medicine King's College London London UK; ^5^ South London & Maudsley NHS Foundation Trust London UK

**Keywords:** cannabidiol, cannabis, complex regional pain syndromes, hyperalgesia, pain, tetrahydrocannabinol, trauma

## Abstract

**Background:**

Complex regional pain syndrome is characterized by severe, persistent pain. Emerging evidence suggests that cannabis‐based medicinal products may represent a new therapeutic option. However, to date, no clinical studies have evaluated the effects of cannabis‐based medicinal products in individuals with complex regional pain syndrome. The aim of this study is to assess changes in patient‐reported outcome measures and the prevalence of adverse events associated with cannabis‐based medicinal products prescribed for complex regional pain syndrome.

**Methods:**

This case series assessed changes in patient‐reported outcome measures over 6 months in complex regional pain syndrome patients enrolled in the UK Medical Cannabis Registry. Adverse events were measured and graded using the Common Terminology Criteria for Adverse Events version 4.0.

**Results:**

A total of 64 patients were identified for inclusion. At baseline, pain severity measured by the Brief Pain Inventory Short Form was 6.69 ± 1.42. This improved at 1 (5.85 ± 1.73), 3 (5.91 ± 1.82), and 6 months (6.05 ± 1.72; *p* < 0.050). Participants also reported improvements in severity as measured by the Short Form‐McGill Pain Questionnaire‐2 and pain visual analogue scale at the same time points (*p* < 0.050). Participants also reported improvements in anxiety symptoms, sleep quality, and general health‐related quality of life (*p* < 0.050), as measured by validated measures. Five patients (7.81%) reported 50 (78.13%) adverse events.

**Discussion:**

This study represents the outcomes in individuals with complex regional pain syndrome prescribed cannabis‐based medicinal products. These suggest initiation of cannabis‐based medicinal products is associated with improvements in patient‐reported outcome measures. While these findings are consistent with the literature, they must be interpreted with caution, considering the limitations of this study.

**Conclusion:**

Cannabis‐based medicinal products were associated with improvements in pain severity and interference. Participants also reported improvements in important metrics of health‐related quality of life. This supports further research through high‐quality randomized controlled trials to ascertain the efficacy of cannabis‐based medicinal products in improving complex regional pain syndrome symptoms.

## Graphical abstract

Complex regional pain syndrome is a rare neuropathic pain disorder. There is a paucity of effective analgesia available for this condition. This study aimed to assess the outcomes of 64 patients prescribe cannabis‐based medicinal products for this condition up to 6 months.

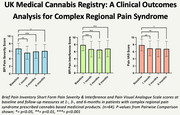

## 1 Background

Complex regional pain syndrome is a condition characterized by severe, chronic neuropathic pain (Dey et al. 2023, [Link], NHS 2022, Ferraro et al. 2024). This pain is disproportionate to the initial trauma and persists beyond the expected healing time (Dey et al. 2023). Complex regional pain syndrome is estimated to affect 5 in 10,000 people in the UK, or 26.2 per 100,000‐person years (Ferraro et al. 2024, de Mos et al. 2007). These figures, however, may underestimate the true scale, as complex regional pain syndrome is commonly underdiagnosed or misdiagnosed (Elsamadicy et al. 2018).

While the precise pathophysiology of complex regional pain syndrome remains elusive, proposed theories implicate abnormal inflammatory processes and mechanisms involved with maladaptive neuroplasticity, which cause heightened sensitization and hyperactivity of the central and peripheral nervous system (Ferraro et al. 2024, Thoma et al. 2022). However, the exact biological pathways initiating and perpetuating complex regional pain syndrome have yet to be fully delineated.

Pharmacological therapies such as nonsteroidal anti‐inflammatory drugs (NSAIDs), gabapentinoids, antidepressants, and opioids are routinely prescribed for complex regional pain syndrome (NaN 2023, Ferraro et al. 2023). However, there is a paucity of high‐quality evidence in the literature supporting the long‐term safety and efficacy of these treatments for chronic neuropathic pain (Hylands‐White et al. 2017, Cohen et al. 2021). A Cochrane review found available evidence on the efficacy and safety of NSAIDs in neuropathic pain to be of low quality, therefore raising concerns about their suitability for complex regional pain syndrome (Moore et al. 2015). Additionally, long‐term use of NSAIDs may confer dose‐dependent adverse events (AEs) in patients, such as gastrointestinal ulcers or cardiovascular events (MacHado et al. 2021, Fine 2013). Similarly, while gabapentinoids are widely used for neuropathic pain, recent evidence indicates low to moderate certainty of their efficacy (Ferraro et al. 2024, Ferraro et al. 2023, Di Stefano et al. 2021). Moreover, gabapentinoids are increasingly associated with potential misuse and dependence from prolonged use (Mcneilage et al. 2023, Goodman and Brett 2017). Opioids are also used in routine practice (Dey et al. 2023, Ferraro et al. 2024). There is, however, insufficient evidence of their effectiveness in improving chronic pain and growing evidence of associated harms (Hylands‐White et al. 2017, Di Stefano et al. 2021, Busse et al. 2018). These include well‐documented risks of opioid dependence and toxicity (Degenhardt et al. 2019). With 20%–40% of patients with complex regional pain syndrome experiencing pain refractory to first‐line pharmacological therapies, there is an urgent need for novel therapeutics for chronic pain secondary to complex regional pain syndrome (Elsamadicy et al. 2018).

There is increasing interest in the endocannabinoid system as a target for complex regional pain syndrome therapy. Cannabinoid type 1 (CB1) receptors are predominantly located within the nervous system and inhibit synaptic transmission of neurotransmitters (Munro et al. 1993, Maldonado et al. 2016). Activation of the CB1 receptor causes potassium efflux and blocks calcium influx, leading to reduced neurotransmitter release from presynaptic terminals (Maldonado et al. 2016, Vučkovic et al. 2018). This mechanism is partially responsible for the analgesic effects of the CB1 receptor agonist anandamide (AEA) (Vučkovic et al. 2018, Hill et al. 2017). Comparatively, cannabinoid type 2 (CB2) receptors, predominantly expressed by immune cells, microglia, and keratinocytes, play a key role in regulating the release of inflammatory cytokines and endogenous opioids (Maldonado et al. 2016, Rahn and Hohmann 2009). Both receptors are upregulated in response to peripheral nerve damage and have been implicated in affecting pain transmission and perception (Lim et al. 2003, Zhang et al. 2003, Hsieh et al. 2011).

Cannabis‐based medicinal products (CBMPs) consist of phytocannabinoids, such as (−)‐*trans*‐Δ^9^‐tetrahydrocannabinol (THC) and cannabidiol (CBD). These are an emerging therapeutic option for complex regional pain syndrome (Vučkovic et al. 2018). THC is a partial agonist of CB1/2 receptors (Pertwee 2008). CBD, meanwhile, causes an increase in the endocannabinoids AEA and 2‐Arachidonoylglycerol (2‐AG) (Vučkovic et al. 2018). CBD binds to fatty acid binding proteins, preventing the intracellular transportation and subsequent degradation of AEA by fatty acid amide hydrolase (Deutsch 2016). Furthermore, CBD also inhibits the breakdown of 2‐AG at synaptic junctions (Vučkovic et al. 2018). Conversely, CBD also acts as a negative allosteric modulator at CB1, reducing the efficacy and potency of THC and endocannabinoids (Laprairie et al. 2015). CBD also acts as an antagonist at G protein‐coupled receptor 55, a G‐protein subunit involved in nociception (Ryberg et al. 2007). Moreover, CBD is postulated to potentiate serotonin via allosteric modulation at the serotonin 1A receptor in the medial prefrontal cortex, leading to the anxiolytic effect of CBMPs (Linge et al. 2016).

Activation of CB2 receptors is proposed to reduce cytokine and chemokine release as well as immune cell migration, hence inhibiting inflammatory processes, a key component of pain sensitization (Vučkovic et al. 2018). CB2 agonism on keratinocytes upregulates the production of beta‐endorphins and enkephalins, which bind to μ‐opioid receptors, decreasing nociception (Vučkovic et al. 2018, Ibrahim et al. 2005). Furthermore, electrophysiology experiments have confirmed colocalization of CB2 and transient receptor potential cation channel subfamily vanilloid member 1 (TRPV1) receptor (González‐Ramírez et al. 2017). CB2 agonists cause downstream inhibition of the adenyl cyclase‐cyclic adenosine monophosphate pathway, preventing phosphorylation of TRPV1, reducing its sensitivity to noxious stimuli (Anand et al. 2020, 2008).

Despite promising preclinical data, there is a paucity of high‐quality evidence evaluating the efficacy and safety of CBMPs for chronic pain, and specifically complex regional pain syndrome (Ferraro et al. 2024, Moore et al. 2021). While a recent Cochrane review demonstrated CBMPs’ association with pain reduction, the evidence was low to moderate quality, and there was significant heterogeneity between studies (Mücke et al. 2018). A meta‐analysis conducted by Wang et al. (2021) found moderate‐ to high‐certainty evidence that non‐inhaled CBMPs confer small improvements in pain relief, physical functioning, and sleep quality in patients with chronic pain. Notably, the latter meta‐analysis did not evaluate inhaled or vaporized forms of CBMPs at all, while the Cochrane review only included two studies comparing inhaled cannabis with placebo, hence limiting their generalizability to other routes of delivery. Notably, up to 60% of patients in the UK are prescribed inhaled CBMPs in the UK to manage chronic pain (Bapir et al. 2023). To address limitations of presently available evidence, the UK Medical Cannabis Registry (UKMCR) was set up to capture real‐world data in 2019 (J. Tait, Erridge, Sodergren, et al. 2023). Prior observational studies based on the UKMCR report that CBMPs are associated with improved health‐related quality of life with a favorable safety profile (Olsson et al. 2023, J. Tait, Erridge, Holvey, et al. 2023). A cohort of patients with chronic pain reported improvements in pain severity across a 6‐month time period compared to baseline when treated with CBMPs (J. Tait, Erridge, Holvey, et al. 2023).

Crucially, there are no existing studies that evaluate the efficacy and safety of CBMPs in a cohort of individuals exclusively with complex regional pain syndrome (Ferraro et al. 2024). CBMPs may confer promising improvements in pain relief and health‐related quality of life for patients with complex regional pain syndrome experiencing pain refractory to current therapies; however, there is a need for novel scientific approaches to address the paucity of high‐quality data. The primary aim of this study was to assess changes in pain‐specific patient‐reported outcome measures (PROMs) in complex regional pain syndrome patients across 6 months of CBMP therapy. Secondary aims include assessment of changes in sleep, anxiety, and general health‐related quality of life, and incidence of AEs.

## 2 Methods

### 2.1 Study Design

This case series analyzed data from the UKMCR. Primary outcomes measured were changes from baseline in validated PROMs following initiation of CBMP treatment. Secondary outcomes included the incidence of AEs. This study followed the Strengthening the Reporting of Observational Studies in Epidemiology guidelines for reporting observational studies (Von Elm et al. 2007).

Ethical approval was provided by the Anonymized Ethics Committee (reference: Anonymized). Patients were enrolled consecutively and provided fully informed written consent.

### 2.2 Settings and Participants

The UKMCR is a privately owned registry designed to collect clinical data on patients prescribed CBMPs through Curaleaf Clinic. The registry's priorities are focused on patient contribution and tailoring its design to capture the impact of CBMPs on health‐related quality of life and condition‐specific outcomes. Patients and clinicians can update comorbidities, medications, AEs, and PROMs using an online data collection portal. A previous evaluation of the registry revealed that high proportions of patients used the electronic portal to fill out questionnaires (92.3%), and participants reported that the portal was easy to use (J. Tait, Erridge, Sodergren, et al. 2023).

Patients enrolled in the UKMCR and treated for a primary indication for complex regional pain syndrome were identified for inclusion in this study. Participants with complex regional pain syndrome were excluded if complex regional pain syndrome was not documented as the primary reason for treatment with CBMPs or if they were enrolled less than 6 months prior to the date of data extraction.

### 2.3 Data Collection/Outcomes

Data was collected at baseline on relevant demographic, medical, and drug and alcohol variables by treating physicians. Demographic variables included age, gender, and occupation, categorized using the Standard Classification of Occupations (International Labour Organization). Additionally, patients’ medication history and comorbidities were recorded and used to calculate a Charlson Comorbidity Index for each patient (Charlson et al. 1987, Quan et al. 2011). Tobacco status and pack years were recorded alongside weekly alcohol consumption. Cannabis use history was documented by classifying patients as cannabis naïve (never having used cannabis), ex‐users, or current users. For ex‐users and current users, cannabis‐gram years were calculated to quantify lifetime exposure (Erridge et al. 2021).

Patients’ medication could be updated using a bespoke online platform or in clinic appointments (J. Tait, Erridge, Sodergren, et al. 2023). Details of the prescribed CBMP formulations were documented, detailing the route of administration and doses of THC & CBD. Formulations included medium‐chain triglyceride oils or dried flower CBMPs. Each type of product was prescribed at the discretion of the treating physician according to clinical need. Due to the observational nature of the study, prescription doses reflected prescriptions issued by members of the General Medical Council's Specialist Register. The choice of product and dose was based on clinical experience, and the reason why each product may be prescribed was not captured.

PROMs, considered the gold standard for assessment of chronic pain and other important domains of health‐related quality of life (Table 1), were collected at baseline, 1, 3, and 6 months via a bespoke electronic reporting tool (Dansie and Turk 2013).

**TABLE 1:** Summary of patient‐reported outcome measures (PROMs) investigated.

Patient‐reported outcome measure	Description	Score	Minimal clinically important difference (MCID)
Brief Pain Inventory (BPI) short form (MD Anderson Cancer Center n.d.)	Assesses both pain intensity and the degree to which pain interferes with various aspects of daily functioning: 1. Pain Severity—measured from a numerical rating scale or the mean of four severity items. 2. Pain Interference—measured as the mean of seven numerical rating scales.	0–10	Raw scores change of 1 point (Dworkin et al. 2008).
Short Form McGill Pain Questionnaire‐2 (SF‐MPQ‐2) (Katz and Melzack n.d.)	Pain severity is assessed across 22 descriptors comprising four subscales: 1. Continuous pain 2. Intermittent pain 3. Neuropathic pain 4. Affective descriptors Each domain is rated from 0 = “*none*” to 10 = “*worst possible*.” A total SF‐MPQ‐2 score is calculated using the mean of all subscales	0–78	Raw scores change of 1 point (Dworkin et al. 2008).
Pain Visual Analogue Scale (VAS) (Haefeli and Elfering 2006)	Assesses pain severity using a 10 cm horizontal line with endpoints; “no pain” at 0 cm and “worst pain imaginable” at 10 cm. The distance from the “no pain” end to the patient's mark is measured in centimeters, providing a pain intensity score out of 10.	0–10	Raw scores change of 1 cm (Dworkin et al. 2008).
European Quality‐of Life 5 Dimension—5 Level (EQ‐5D‐5L) (Hernandez et al. 2019)	Assesses the quality of life across 5 key domains: 1. Mobility 2. Self‐care 3. Unusual activities 4. Pain and discomfort 5. Anxiety and depression For each domain the individual selects one of five levels: no problems, slight problems, moderate problems, severe problems, or extreme problems/unable to. The values obtained are used to form an index score.	−0.594 to 1	n/a
Patient Global Impression of Change (PGIC) (Ferguson and Scheman 2009)	Assesses individuals’ overall impression of change in their condition or symptoms using 7‐point numerical scale. Lower scores indicate no change/worsening and higher scores indicate greater improvement.	1–7	n/a
Generalized Anxiety Disorder‐7 (GAD‐7) (Spitzer et al. 2006)	Screen for and assess the severity of generalized anxiety disorder symptoms corresponding to the Diagnostic and Statistical Manual of Mental Disorders. Individuals rate how often they experience several symptoms on a 4‐point numerical scale from 0 (*not at all*) to 3 (*nearly every day*). Values are added to give a total score. Higher scores indicate greater anxiety severity	0–21	Score change of 4 points (Toussaint et al. 2020).
Single‐Item Sleep Quality Scale (SQS) (Snyder et al. 2018)	Participants are asked to rate their sleep quality over the past 7 days across a scale from 0 to 10. In this evaluation 0 and 10 represent terrible and excellent sleep quality respectively.	0–10	Increase of 2.6 points (Snyder et al. 2018).

*Note*: Yellow shading = Pain‐Specific PROMs. Blue shading = Health‐related Quality of Life PROMs.

Abbreviation: n/a, not applicable.

The minimal clinically important difference (MCID) values used in this study correspond to published values in the literature for collected PROMs (Table 1) (Dworkin et al. 2008).

AEs were reported in accordance with the Common Terminology Criteria for AEs version 4.0 (National Cancer Institute 2009, Trotti et al. 2016).

### 2.4 Missing Data

Missing data was accounted for by using the Baseline Observation Carried Forward Approach (BOCF), as recommended by the European Medicines Agency (Committee for Medicinal Products for Human Use (CHMP) 2010). This method assumes no positive benefit is observed from CBMP treatment, providing a conservative estimate of CBMPs’ efficacy (Liu‐Seifert et al. 2010).

### 2.5 Statistical Analysis

Descriptive statistics were used to analyze demographic variables as well as tobacco, alcohol, and cannabis status data. Statistical tests were performed to determine significance and to construct data tables. Frequency was presented as *n* (%). Parametric data was presented as mean (standard deviation [SD]), with nonparametric data presented as median (interquartile range [IQR]). *p* < 0.050 was considered statistically significant.

Differences in PROMs were assessed using a repeated measures analysis of variance (ANOVA). Post hoc analyses with Bonferroni correction were conducted on values, which were statistically significant to identify individual differences between each time period.

Univariable and multivariable logistic regression analyses were planned to determine the relationship between individual variables and the likelihood of a clinically significant improvement in pain severity based on published MCID for Brief Pain Inventory (BPI) Pain Severity, Short Form‐McGill Pain Questionnaire‐2 (SF‐MPQ‐2) Overall Score, and Pain Visual Analogue Scale (VAS). Data were presented as odds ratios (OR) with 95% confidence intervals (95% CI).

Data analyses were performed using IBM Statistical Package for Social Sciences (SPSS) version 29 (IBM Corp 2023).

## 3 Results

### 3.1 Patient Demographic Data

**FIGURE 1:**
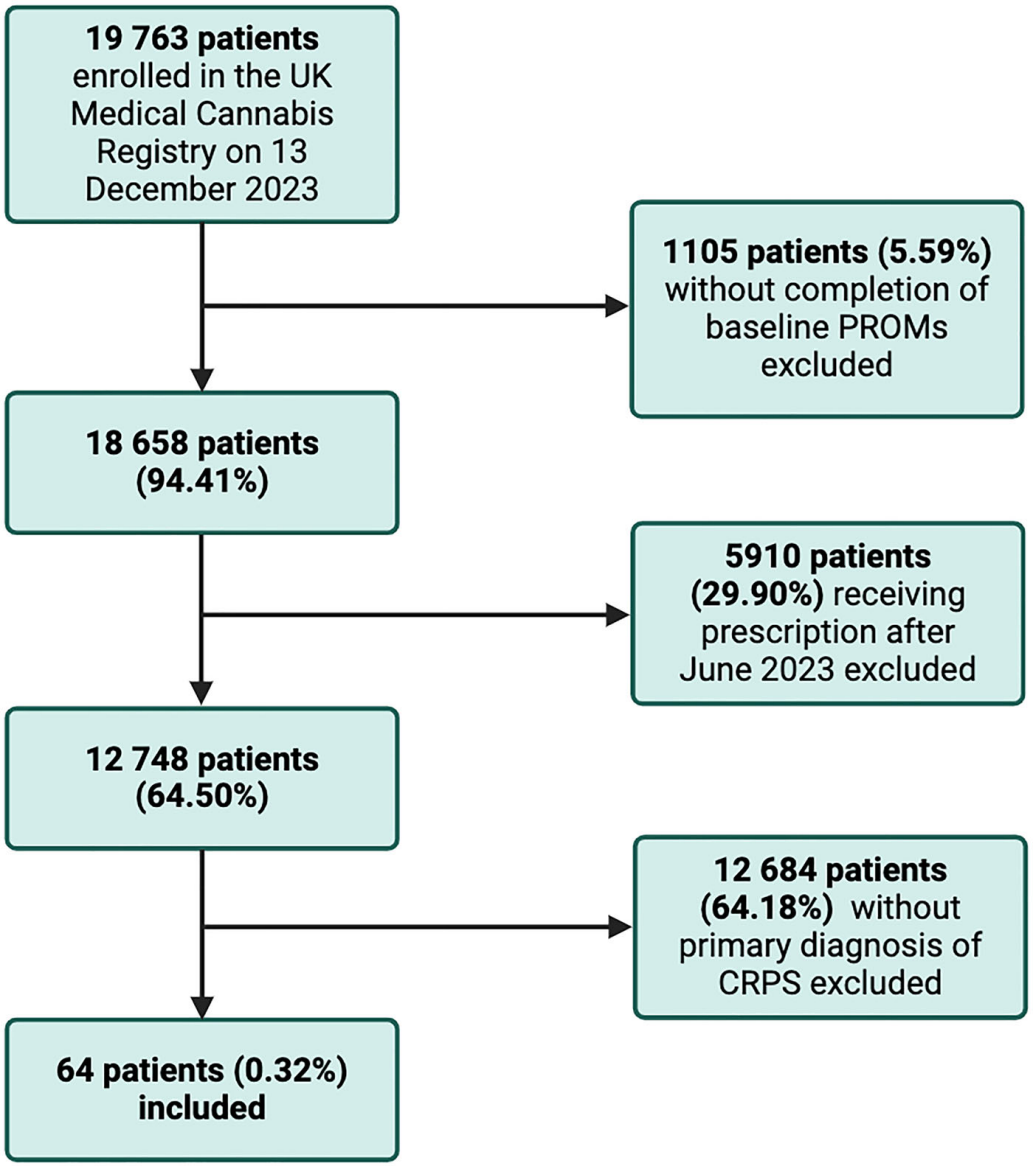
Flowchart illustrating data inclusion and exclusion. CRPS, Complex regional pain syndrome; PROMs, patient‐reported outcome measures.

Patient data was extracted from the UKMCR, identifying 64 patients with a primary diagnosis of complex regional pain syndrome that met the inclusion criteria (Figure 1).

The mean age of patients was 41.91 ± 11.25 years (Table 2). There were 31 (48.44%) female and 33 (51.56%) male patients. Most patients, 32 (50.00%), selected “unemployed” as their occupation. The mean body mass index (BMI) of patients was 26.30 ± 7.04 kg/m^2^.

**TABLE 2:** Table of study participant demographics and tobacco, alcohol, and cannabis status (*n* = 64).

**Demographic Details**		***n* (%)/median [IQR]/mean ± SD**
Gender	Female	31 (48.44)
	Male	33 (51.56)
Age (years)		41.91 ± 11.25
Occupation	Clerical support workers	3 (4.69)
	Craft and related trades workers	3 (4.69)
	Managers	2 (3.13)
	Other occupations	17 (26.56)
	Professional	4 (6.25)
	Retired	1 (1.56)
	Services and sales workers	1 (1.56)
	Technicians and associate professionals	1 (1.56)
	Unemployed	32 (50.00)
Body mass index (kg/m^2^)		26.30 ± 7.04
Charlson comorbidity index		0.00 [0.00–3.50]
Tobacco status and consumption	Current smoker	17 (26.56)
	Ex‐smoker	25 (39.06)
	Never smoked	22 (34.38)
	Pack years	10.00 [5.00–20.00]
Weekly alcohol consumption (units)		0.00 [0.00–15.00]
Cannabis status and consumption	Never used	22 (34.38)
	Ex‐user	6 (9.38)
	Current user	36 (56.25)
	Cannabis consumed per day (grams)	1.50 [1.00–7.50]
Cannabis use frequency	Every day	32 (50.00)
	Every other day	3 (4.69)
	More than one time per month	1(1.56)
	Lifetime quantity of cannabis consumed (gram years)	8.00 [4.50–35.50]

Participants involved in this study were from regions across the United Kingdom (Appendix A in the Supporting Information). A total of 13 (20.30%) patients were from Scotland, 10 (15.60%) were from the West Midlands, and another 10 (15.60%) were from the South East.

### 3.2 Tobacco, Alcohol, and Cannabis Status

Participants’ tobacco, alcohol, and cannabis status is outlined in Table 2. A total of 42 (65.63%) participants were current or ex‐smokers, and the median pack years was reported as 10.00 [5.00–20.00]. A total of 22 (34.38%) participants had never consumed cannabis. A total of 42 (65.63%) patients were current or ex‐users of cannabis, and the median lifetime quantity of cannabis consumed was 8.00 [4.50–35.50] gram years.

### 3.3 Cannabis‐Based Medicinal Products

Table 3 details the CBMP formulation and median daily doses of THC and CBD at baseline and 6 months. The median CBD dose prescribed at baseline and 6 months was 11.00 [1.13–20.00] mg/day and 20.00 [10.00–29.88] mg/day, respectively. The median THC dose prescribed at baseline and 6 months was 12.50 [1.00–21.38] mg/day and 117.86 [29.25–222.15] mg/day, respectively. Most participants were prescribed both oils and dried flower formulations at both baseline and 6 months. Adven EMC1 50/< 4 mg/mL CBD/THC (Curaleaf International, United Kingdom) and Adven EMT 20 mg/mL THC (Curaleaf International, United Kingdom) were the most frequently prescribed CBD‐ and THC‐dominant oils. The most commonly prescribed flos was Adven EMT2 16%/< 1% THC/CBD (Curaleaf International, United Kingdom).

**TABLE 3:** Cannabis‐based medicinal product prescriptions (*n* = 64). Dose presented as median [interquartile range]. Proportions are represented as the number of participants (percentage).

	**Baseline**	**6 months**
CBD dose (mg/day)	11.00 [1.13–20.00]	20.00 [10.00–29.88]
THC dose (mg/day)	12.50 [1.00–21.38]	117.86 [29.25–222.15]
Oils	28 (43.80%)	18 (28.10%)
Dried Flower	6 (9.40%)	8 (12.50%)
Both	30 (49.60%)	38 (59.40%)

### 3.4 Patient‐Reported Outcome Measures

Repeated measures ANOVA tests were performed, displaying the mean scores of pain‐specific and health‐related quality of life PROMs at baseline, 1, 3, and 6 months follow‐up (Appendix B in the Supporting Information). For all pain‐specific PROMs, an improvement was seen between baseline and 6‐month follow‐up (*p *< 0.010). For the health‐related quality of life PROMs, except EQ‐5D‐5L Self‐Care (*p* = 0.866) and PGIC (*p* = 0.424), an improvement was seen between baseline and 6‐month follow‐up (*p* < 0.050).

**FIGURE 2:**
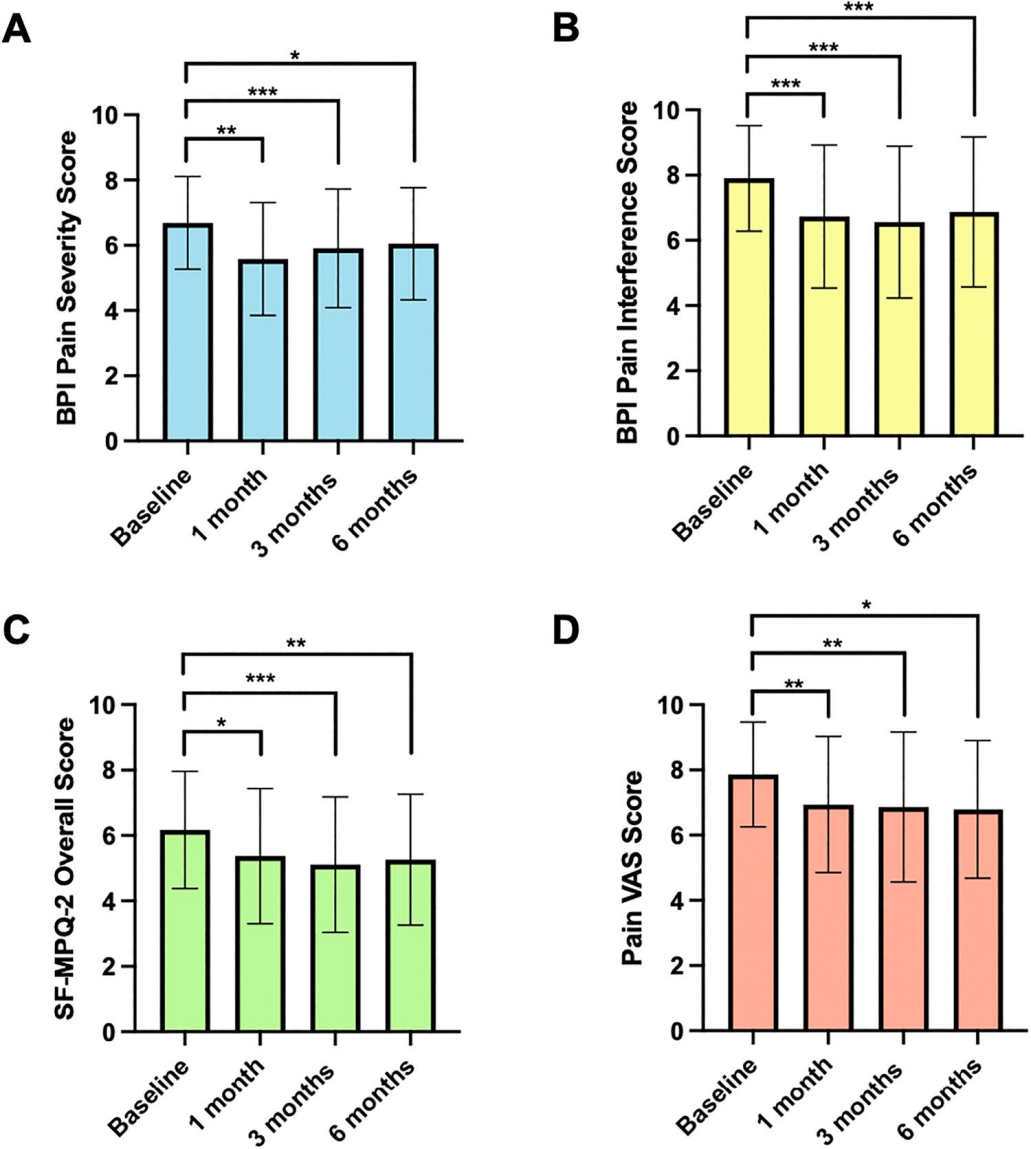
Comparison of pain‐specific patient‐reported outcome measures between baseline, 1, 3, and 6 months. Figure created using GraphPad Prism. (A) Brief Pain Inventory (BPI) pain severity (*n* = 64), (B) BPI pain interference (*n* = 64), (C) Short Form McGill Pain Questionnaire‐2 (SF‐MPQ‐2) Overall Score (*n* = 63), (D) Pain VAS (*n* = 64). *p* values shown; (***p < 0.001, **p < 0.010, *p < 0.050). Any other comparisons are nonsignificant (*p* > 0.050).

Pairwise comparisons were performed on PROMs, which were statistically significant on ANOVA (Table 4). For BPI Pain Severity, BPI Pain Interference, SF‐MPQ‐2 Neuropathic Pain, Affective Descriptors, Overall Score, and Pain VAS PROMs, improvements in scores were observed separately at each follow‐up period compared to baseline scores (Figure 2; *p* < 0.050). However, for SF‐MPQ‐2 Continuous Pain and Intermittent Pain, an improvement was only achieved at 3‐ and 6‐month follow‐up in comparison to baseline (*p* < 0.050). At 6‐months, a significant difference was observed in all pain‐specific PROMs in comparison to baseline (*p* < 0.050). No changes were seen at each follow‐up between 1‐ and 3‐month scores and later follow‐up periods (*p* > 0.050).

**TABLE 4:** Pairwise comparisons of patient‐reported outcome measures at baseline, 1, 3, and 6 months.

	**Baseline**		**1 month**		**3 months**		**6 months**		
**BPI pain severity**	**MD ± SD**	***p* value**	**MD ± SD**	***p* value**	**MD ± SD**	***p* value**	**MD ± SD**		***p* value**
Baseline									
1 month	0.84 ± 1.68	0.001**							
3 months	0.78 ± 1.50	< 0.001***	−0.06 ± 1.34	1.000					
6 months	0.64 ± 1.62	0.015*	−0.20 ± 1.49	1.000	−0.14 ± 1.18	1.000			
**BPI pain interference**	**MD ± SD**	***p* value**	**MD ± SD**	***p* value**	**MD ± SD**	***p* value**	**MD ± SD**		***p* value**
Baseline									
1 month	1.17 ± 2.00	< 0.001***							
3 months	1.34 ± 1.92	< 0.001***	0.17 ± 1.94	1.000					
6 months	1.03 ± 2.00	< 0.001***	−0.15 ± 1.94	1.000	0.31 ± 0.23	1.000			
**SF‐MPQ‐2 neuropathic pain**	**MD ± SD**	***p* value**	**MD ± SD**	***p* value**	**MD ± SD**	***p* value**	**MD ± SD**	***p* value**	
Baseline									
1 month	0.77 ± 1.93	0.015*							
3 months	0.93 ± 1.79	< 0.001***	0.16 ± 1.93	1.000					
6 months	0.93 ± 2.06	0.004**	0.16 ± 2.03	1.000	0.00 ± 1.71	1.000			
**SF‐MPQ‐2 affective descriptors**	**MD ± SD**	***p* value**	**MD ± SD**	***p* value**	**MD ± SD**	***p* value**	**MD ± SD**	***p* value**	
Baseline									
1 month	1.08 ± 2.61	0.010*							
3 months	1.33 ± 2.52	< 0.001***	0.26 ± 2.31	1.000					
6 months	1.14 ± 2.32	0.001**	0.06 ± 2.02	1.000	−0.19 ± 2.17	1.000			
**SF‐MPQ‐2 continuous pain**	**MD ± SD**	***p* value**	**MD ± SD**	***p* value**	**MD ± SD**	***p* value**	**MD ± SD**	***p* value**	
Baseline									
1 month	0.73 ± 2.31	0.093							
3 months	0.79 ± 1.85	0.007**	0.07 ± 1.95	1.000					
6 months	0.80 ± 2.03	0.017*	0.07 ± 1.99	1.000	0.01 ± 1.80	1.000			
**SF‐MPQ‐2 intermittent pain**	**MD ± SD**	***p* value**	**MD ± SD**	***p* value**	**MD ± SD**	***p* value**	**MD ± SD**	***p* value**	
Baseline									
1 month	0.62 ± 2.13	0.146							
3 months	1.17 ± 2.04	< 0.001***	0.55 ± 2.29	0.360					
6 months	0.80 ± 1.92	0.010*	0.18 ± 1.91	1.000	−0.38 ± 1.91	0.240			
**SF‐MPQ‐2 overall score**	**MD ± SD**	***p* value**	**MD ± SD**	***p* value**	**MD ± SD**	***p* value**	**MD ± SD**	***p* value**	
Baseline									
1 month	0.80 ± 1.93	0.010*							
3 months	1.06 ± 1.73	< 0.001***	0.26 ± 1.79	1.000					
6 months	0.92 ± 1.85	0.001**	0.12 ± 1.60	1.000	−0.14 ± 1.60	1.000			
**Pain VAS**	**MD ± SD**	***p* value**	**MD ± SD**	***p* value**	**MD ± SD**	***p* value**	**MD ± SD**	***p* value**	
Baseline									
1 month	0.92 ± 2.14	0.007**							
3 months	1.00 ± 2.10	0.002**	0.08 ± 1.99	1.000					
6 months	0.89 ± 2.22	0.014*	−0.03 ± 1.75	1.000	−0.11 ± 1.87	1.000			
**EQ‐5D‐5L index**	**MD ± SD**	***p* value**	**MD ± SD**	***p* value**	**MD ± SD**	***p* value**	**MD ± SD**	***p* value**	
Baseline									
1 month	−0.21 ± 0.23	< 0.001***							
3 months	−0.18 ± 0.25	< 0.001***	0.03 ± 0.20	1.000					
6 months	−0.17 ± 0.26	< 0.001***	0.04 ±0.19	1.000	0.01 ± 0.20	1.000			
**EQ‐5D‐5L mobility**	**MD ± SD**	***p* value**	**MD ± SD**	***p* value**	**MD ± SD**	***p* value**	**MD ± SD**	***p* value**	
Baseline									
1 month	0.27 ± 0.67	0.015*							
3 months	0.17 ± 1.02	1.000	−0.09 ± 0.83	1.000					
6 months	0.27 ± 0.78	0.051	0.00 ± 0.69	1.000	0.09 ± 0.92	1.000			
**EQ‐5D‐5L usual activities**	**MD ± SD**	***p* value**	**MD ± SD**	***p* value**	**MD ± SD**	***p* value**	**MD ± SD**	***p* value**	
Baseline									
1 month	0.45 ± 0.89	< 0.001 ***							
3 months	0.28 ± 0.94	0.113	−0.17 ± 0.81	0.562					
6 months	0.30 ± 0.89	0.056	−0.16 ± 0.96	1.000	0.02 ± 0.86	1.000			
**EQ‐5D‐5L pain and discomfort**	**MD ± SD**	***p* value**	**MD ± SD**	***p* value**	**MD ± SD**	***p* value**	**MD ± SD**	***p* value**	
Baseline								***p* value**	
1 month	0.78 ± 0.97	< 0.001 ***							
3 months	0.77 ± 0.87	< 0.001***	−0.02 ± 0.81	1.000					
6 months	0.58 ± 0.98	< 0.001***	−0.20 ± 0.82	0.311	−0.19 ± 0.09	0.305			
**EQ‐5D‐5L anxiety and depression**	**MD ± SD**	***p* value**	**MD ± SD**	***p* value**	**MD ± SD**	***p* value**	**MD ± SD**	***p* value**	
Baseline									
1 month	0.55 ± 1.01	< 0.001***							
3 months	0.42 ± 1.14	0.026*	−0.13 ± 0.74	1.000					
6 months	0.41 ± 1.10	0.025*	−0.14 ± 0.78	0.906	−0.02 ± 0.81	1.000			
**GAD‐7**	**MD ± SD**	***p* value**	**MD ± SD**	***p* value**	**MD ± SD**	***p* value**	**MD ± SD**	***p* value**	
Baseline									
1 month	3.28 ± 5.34	< 0.001***							
3 months	2.03 ± 5.83	0.042*	−1.25 ± 4.10	0.106					
6 months	2.17 ± 5.03	0.006**	−1.11 ± 4.34	0.270	0.14 ± 3.29	1.000			
**SQS**	**MD ± SD**	***p* value**	**MD ± SD**	***p* value**	**MD ± SD**	***p* value**	**MD ± SD**	***p* value**	
Baseline									
1 month	−1.23 ± 2.66	0.003**							
3 months	−1.39 ± 2.54	< 0.001***	−0.16 ± 1.95	1.000					
6 months	−1.13 ± 2.48	0.003**	0.11 ± 2.01	1.000	0.27 ± 1.80	1.000			

*Note*: Pairwise comparison with Bonferroni correction was performed in IBM Statistical Package for Social Sciences (SPSS) version 29. BPI Pain Severity and Interference, Pain VAS, EQ‐5D‐5L, GAD‐7, PGIC, and SQS. (*n* = 64). SF‐MPQ‐2 Neuropathic pain, affective descriptors, continuous pain, intermittent pain and overall score (*n* = 63). *p* values shown; (***p < 0.001, **p < 0.010, *p < 0.050). Green shading = *p* < 0.050. Red shading = *p* ≥ 0.05.

Abbreviation: MD ‐ mean difference; SD ‐ standard deviation

Improvements were recorded between baseline and each follow‐up period for EQ‐5D‐5L Index Value, EQ‐5D‐5L Pain and Discomfort, EQ‐5D‐5L Anxiety and Depression, GAD‐7, and SQS (*p* < 0.050). For EQ‐5D‐5L Mobility (*p* = 0.0150) and Self‐Care (*p *< 0.001), an improvement was only seen at 1 month in comparison to baseline. There were no differences in the pairwise comparison of 1‐, 3‐, and 6‐month values (*p* > 0.050).

### 3.5 Minimal Clinically Important Differences

Improvements in pain severity PROMs corresponded to published clinically important thresholds. MCID was achieved in 18 (28.10%) of patients for BPI pain severity, 26 (40.6%) for SF‐MPQ‐2 overall score, and 24 (37.50%) in Pain VAS at 6 months.

### 3.6 Logistic Regression

Previous use of illicit cannabis was associated with a MCID improvement in Pain VAS scores at 6‐month follow‐up on both univariate (OR: 6.00; 95% CI, 1.53–23.46; *p* = 0.010) and multivariate logistic regression (OR: 28.63; 95% CI, 2.53–324.51; *p* = 0.007). On multivariate analyses an MCID in BPI severity was associated with being over the age of 40 (OR: 7.79; 95% CI, 1.40–43.32; *p* = 0.019). For all other variables investigated, no associations with MCID were demonstrated on univariate or multivariate binary regression (Appendices C and D in the Supporting Information).

### 3.7 Adverse Events

Five patients (7.81%) of patients reported a total of 50 (78.13%) AEs. Of the AEs experienced, 15 (30.00%) were classified as mild, 15 (30.00%) as moderate, and 20 (40.00%) as severe (Table 5). The most common AEs experienced were nausea (*n* = 4; 6.25%) and headache (*n* = 4; 6.25%).

**TABLE 5:** Adverse Events reported by patients prescribed cannabis‐based medicinal products for complex regional pain syndrome (*n* = 64).

**Adverse events**	**Mild**	**Moderate**	**Severe**	**Life‐threatening/disabling**	**Total (%)**
Abdominal pain	2	1	0	0	3 (4.69)
Anorexia	1	0	0	0	1 (1.56)
Blurred vision	0	0	2	0	2 (3.13)
Cognitive disturbance	0	0	2	0	2 (3.13)
Concentration impairment	0	0	2	0	2 (3.13)
Confusion	1	1	0	0	2 (3.13)
Constipation	3	0	0	0	3 (4.69)
Delirium	1	0	0	0	1 (1.56)
Dizziness	0	1	2	0	3 (4.69)
Dry mouth	1	0	0	0	1 (1.56)
Dyspepsia	1	2	0	0	3 (4.69)
Fatigue	1	0	2	0	3 (4.69)
Generalized muscle weakness	0	0	2	0	2 (3.13)
Headache	0	2	2	0	4 (6.25)
Insomnia	0	0	2	0	2 (3.13)
Lethargy	0	2	0	0	2 (3.13)
Nausea	4	0	0	0	4 (6.25)
Pharyngitis	0	1	0	0	1 (1.56)
Somnolence	0	1	2	0	3 (4.69)
Tremor	0	1	0	0	1 (1.56)
Urinary tract pain	0	2	0	0	2 (3.13)
Vertigo	0	0	2	0	2 (3.13)
Vomiting	0	1	0	0	1 (1.56)
**Total**	**15**	**15**	**20**	**0**	**50 (78.13)**

## 4 Discussion

This case series represents the first analysis of clinical evidence demonstrating an association between initiation of CBMP treatment specifically for complex regional pain syndrome and patient‐reported improvements in pain severity. Improvements between baseline and all follow‐up scores were also observed with all other health‐related quality of life PROMs, except EQ‐5D‐5L Self‐Care and PGIC. However, a considerable proportion of patients did not achieve an MCID in BPI Pain Severity, SF‐MPQ‐2 Overall Score, or Pain VAS. Clinically significant improvements in SF‐MPQ‐2 Overall Score were independent of patient factors, such as age, gender, BMI, cannabis status, and route of CBMP administration. Patients who were ex‐ or current users of illicit cannabis prior to the study were more likely to experience a clinically significant improvement in their Pain VAS scores as seen on univariate and multivariate regression.

This study observed improvements in pain‐specific PROMs following the initiation of CBMP treatment. These findings are consistent with existing literature which similarly demonstrates an association between CBMP treatment and consistent improvements in pain severity in chronic or neuropathic pain conditions (Wang et al. 2021, J. Tait, Erridge, Holvey, et al. 2023, Wilsey et al. 2013, 2008, Abelev et al. 2022, Ware et al. 2010, Rog et al. 2005). Two randomized, double‐blind, placebo‐controlled randomized controlled trials (RCTs) conducted by Wilsey et al. reported improvements in pain severity across a heterogeneous neuropathic pain population, including individuals with complex regional pain syndrome (Wilsey et al. 2013, 2008). These studies utilized Pain VAS like the present analysis. However, while PGIC scores indicated improvements in symptoms for patients, there were no statistically significant improvements over time on ANOVA, unlike Wilsey et al. (2013), who reported significant improvements using Tukey's Honest Significant Difference Test. Notably, Wilsey et al. included patients with a broad spectrum of neuropathic pain conditions (Wilsey et al. 2013, 2008). Limiting the population to individuals with complex regional pain syndrome in the present study helps to reduce potential confounding of response in other conditions. Moreover, there is ongoing contention in the literature as to whether complex regional pain syndrome should be included in neuropathic pain disorders due to the condition's unique pathophysiology associated with different subtypes, therefore raising concerns about the generalizability of neuropathic pain studies to complex regional pain syndrome (Naleschinski and Baron 2010). Extrapolating and comparing findings from neuropathic pain studies must be done with caution and this knowledge in mind.

A cross‐sectional analysis of CBMPs for chronic refractory pain by Abelev et al. (2022) did not report statistically significant differences in pain‐severity scores, despite observing improvements in other PROMs over a 4‐month period. However, in contrast to the findings of the present study, a higher proportion of patients (32.9%) achieved a MCID in pain‐intensity outcomes, compared to 28.10% of patients achieving a MCID in BPI Severity in the present study (Abelev et al. 2022). This could be attributed to discrepancies in methods, such as different PROM selections and definitions of MCID. Abelev et al. (2022) utilized Patient Reported Outcome Measures Information System 29, hence yielding different results. Likewise, the Quality of Life Evaluation Study (QUEST) initiative also reported a higher proportion of patients achieving the MCID compared to the present study's findings (M. A. Tait, Costa, et al. 2023). This study utilized the European Organization for Research and Treatment of Cancer Quality of Life questionnaire (QLQ‐C30) for pain assessment (M. A. Tait, Costa, et al. 2023). The use of the BPI, SF‐MPQ‐2 and Pain VAS in the present study are better recognized assessments for pain severity, as highlighted by the Initiative on Methods, Measurement, and Pain Assessment in Clinical Trials (Dworkin et al. 2008). Reasons for the discrepancy in the results could be secondary to the use of the BOCF methods for accounting for missing data in the present study, biasing the study towards a null finding. Additionally, the 6‐months follow‐up of the present study was longer than the compared studies, which may indicate potential tolerance to the effects of CBMPs over time.

While the observational nature of this study did not enable comparison of CBMPs with other complex regional pain syndrome pharmaceutical therapies, there is evidence in the literature to support their use over current therapeutic options (Di Stefano et al. 2021, Takakuwa et al. 2020, Nutt et al. 2022). Jeddi et al. (2024)’s network meta‐analysis concluded that CBMPs have a good benefit‐safety profile compared to other treatments for chronic neuropathic pain including opioids, owing to its similar efficacy and lower discontinuation rates secondary to AEs. Importantly, the observed changes in pain‐specific PROMs in this study may confer opioid‐sparing effects in complex regional pain syndrome patients. A UKMCR prospective cohort study by Bapir et al. (2023) reported that patients with chronic pain who initiated medicinal cannabis treatment experienced a reduction in opioid use, which is mirrored by the results of other studies (Vigil et al. 2017, Corroon et al. 2017). Previously, complex regional pain syndrome patients have been treated with opioid analgesics, which are associated with a range of adverse effects, including respiratory depression, constipation, and the risk of dependence (Dey et al. 2023, Ferraro et al. 2024, Degenhardt et al. 2019). While opioids are not recommended in complex regional pain syndrome, they are often prescribed due to a paucity of available therapies supported by high‐quality research (Dey et al. 2023, Ferraro et al. 2024, Andreas et al. 2012). Future research on complex regional pain syndrome patients from the UKMCR should aim to evaluate changes in prescribed analgesics, including opioids.

This study found that CBMP treatment was associated with improvements in health‐related quality of life, mirroring the results of similar studies utilizing data from the UKMCR (J. Tait, Erridge, Holvey, et al. 2023, Abelev et al. 2022, M. A. Tait, Costa, et al. 2023, Rapin et al. 2021, Erridge et al. 2023, Kelley et al. 2024). This is a notable finding as complex regional pain syndrome is known to have detrimental impacts on patients’ overall quality of life (Dey et al. 2023). Assessment of health‐related quality of life is an additional metric for assessing CBMPs’ clinical impact as significant improvements in pain are known to positively influence physical, emotional, and social functioning (Turk et al. 2016). However, improvements in pain severity do not always correlate to improvements in functional abilities, reflecting the biopsychosocial nature of complex regional pain syndrome (Llewellyn et al. 2019, Finnmann Munk et al. 2022). Unlike previous studies, the present study did not identify an improvement in EQ‐5D‐5L Self Care scores at follow‐up in comparison to baseline. This may be due to deconditioning, motor learning deficits, fear of movement, and avoidance behaviors associated with long‐term chronic pain that are cited in the literature (Meints and Edwards 2018). In contrast, Wang et al. (2021)’s systematic review and meta‐analysis of RCTs investigating medical cannabis for chronic pain found moderate to high‐certainty evidence to imply that CBMPs do not improve emotional or social functioning. Although this review did not include studies using vaporized or inhaled forms of medical cannabis, limiting its generalizability to the results of the present study. Furthermore, heterogeneity in cannabis formulations and route of administration can influence pharmacokinetic interactions and metabolism producing, variations in observed health‐related quality of life outcomes (Lucas et al. 2018, Stella et al. 2021). Moreover, while improvements were seen in GAD‐7 scores from baseline and each follow‐up in the present study, the classified severity of the mean scores remained as “mild anxiety” throughout. Addressing both pain perception and nociceptive transmission are important in improving chronic pain patients’ health‐related quality of life.

Within the present study, five (7.81%) of patients reported a total of 50 (78.13%) AEs, an incidence lower than cited in previous studies (Bapir et al. 2023, Olsson et al. 2023, J. Tait, Erridge, Holvey, et al. 2023, Abelev et al. 2022). Similar to prior studies, patients who experience one AE were likely to experience multiple AEs (Olsson et al. 2023, Abelev et al. 2022). However, unlike previous studies, a higher proportion of patients experienced severe AEs. Other studies, such as Abelev et al. (2022)’s typically required AEs to be verified, graded, and reported by healthcare professionals, whereas the present study had patients report AEs independent of clinical verification, potentially introducing responder bias. The frequent and thorough requests for patients’ AE data from Curaleaf Clinic could have resulted in overestimations of the incidence and severity of AEs. Notably, psychosis‐related AEs typically associated with illicit use of cannabis, such as paranoia, euphoria, and hallucinations, were not reported by any patients in this study (Childs et al. 2017, Johns 2001). This finding suggests that the monitored use that CBMPs offer may mitigate the risk of these severe adverse reactions.

### 4.1 Limitations

As an observational case series there was an inherent lack of randomization and controlled variables. For example, patients were not standardized at baseline with respect to other medications prescribed. Therefore, the present study is not able to assess whether this affected the study outcomes. Consequently, it is not possible to prove that CBMPs were the cause of the reported findings in the present study.

Additionally, CBMPs are known to have pharmacokinetic interactions with other medications through inhibition of cytochrome P450 enzymes involved in drug metabolism (Lucas et al. 2018). The influence of these drug–drug interactions could have affected the incidence of polypharmacy induced‐AEs. However, evaluation of the influence of concurrent prescriptions was beyond the scope and capabilities of this study. Considering polypharmacy is an important consideration for complex regional pain syndrome patients, future research could also investigate whether CBMP treatment influences the discontinuation of other medications.

Allodynia and hyperalgesia are components of complex regional pain syndrome that were not evaluated in this study. Prior RCTs by Wilsey et al. performed evoked pain assessments after CBMP treatment using a thermode (Wilsey et al. 2013, 2008). Despite PROM scores indicating improvements in pain severity, the thermode test demonstrated that CBMPs analgesic effect did not translate to thermal evoked pain. Future studies could consider potential means of assessing improvements in evoked pain although, important ethical considerations would need to be reviewed.

This study may have introduced both recall and responder bias, particularly concerning patients’ cannabis status. With respect to PROMs, patients are required to provide subjective responses to characterize the severity of each outcome at defined time periods, separated by many months. These are the current gold standard measures of assessing pain, anxiety, and health‐related quality of life. Reliability of these responses could be improved, however, by using ecological momentary assessment (May et al. 2018). Moreover, the development of objective biomarkers or digital assessment tools may help improve this scientific field in the future (Shirvalkar et al. 2023, Vitali et al. 2024). Moreover, due to social desirability bias, patients may not have underreported their prior illicit cannabis use.

In the present study, there was a higher proportion of males compared to the anticipated complex regional pain syndrome population, despite the fact that female are four times more likely to experience complex regional pain syndrome (Ferraro et al. 2024). This is consistent with prior studies, which similarly report a higher proportion of male participants prescribed CBMPs for other conditions (Olsson et al. 2023, Wilsey et al. 2008, Vigil et al. 2017, Ware et al. 2005, Cahill et al. 2021). Additionally, mirroring other studies, over 50% of the present study's participants reported use of illicit cannabis prior to commencing CBMP treatment (Ware et al. 2005, Cahill et al. 2021). This may be explained by recent data from our group which has shown that men are more likely to report illicit cannabis for health reasons compared to women (Erridge et al. 2024).

Both expectancy bias and patients’ preconceived awareness of cannabis’ side effects could have influenced reporting of AEs and PROM improvements, leading to potential overstatements about CBMPs effects. The associated placebo effect may have been enhanced by CBMPs’ psychoactive and vasoactive properties.

Additional limitations include clinician‐selected CBMP treatment and the private clinic setting which could introduce potential selection bias from clinicians. Utilization of de‐identified electronic patient data at clinic appointments as performed by the QUEST Initiative study could mitigate some of this bias (M. A. Tait, Costa, et al. 2023). Employment of this approach enables healthcare professionals to be blinded to patient identities and previous PROM scores and AE reports. This methodological choice would enhance the study's internal validity by minimizing the risk of response bias.

Lastly, the follow‐up period of this study was limited by the paucity of data on the effect of CBMPs on complex regional pain syndrome beyond 6‐months. This restricted long‐term assessment of AEs and the sustainability of PROM improvements. Additionally, the small sample size of the study was insufficient to perform detailed analyses of AEs, limiting the robustness of conclusions. The small sample size is also a limitation of the study in general, despite complex regional pain syndrome being a rare condition. Future studies with larger cohorts and extended follow‐up periods are needed to address these limitations and provide better insights into CBMPs long term safety profile and efficacy. Specific improvements to study design could incorporate ecological momentary assessment to capture daily impact on pain to reduce recall bias, or to include objective markers of outcomes through wearable technology. Ultimately RCTs are necessary. As complex regional pain syndrome is comparatively rare amongst other chronic pain etiologies novel methods for health technology assessment using real‐world data to generate external controls may help improve recruitment for such studies.

## 5 Conclusion

In conclusion, the results imply that initiation of CBMPs was associated with improved pain relief and health‐related quality of life in complex regional pain syndrome patients. However, it must be noted that this study and the wider literature on CBMPs are subject to significant limitations. RCTs or novel approached to real‐world evidence evaluation will be necessary to ultimately determine the utility of CBMPs for complex regional pain syndrome. In this study, clinically important improvements in Pain VAS were associated with previous cannabis use. Low incidence of AEs was reported, although the proportion of severe AEs documented was higher than previously cited in the literature.

## Author Contributions

**Lilia Evans**: study conception and design, acquisition of data, analysis and interpretation of data, drafting of manuscript, critical revision. **Simon Erridge**: study conception and design, acquisition of data, analysis and interpretation of data, drafting of manuscript, critical revision. **Mikael H. Sodergren**: study conception and design, analysis and interpretation of data, drafting of manuscript, critical revision. **Madhur Varadpande**: acquisition of data, critical revision. **Isaac Cowley**: acquisition of data, critical revision. **Arushika Aggarwal**: acquisition of data, critical revision. All authors read and approved the final manuscript.

## Ethics Statement

Provided by South West–Central Bristol Research Ethics Committee (Reference: 22/SW/0145).

## Consent

All study participants gave formal, informed, and written consent, preceding their consecutive enrollment into the database.

## Conflicts of Interest

Lilia Evans is a medical student at Imperial College London. Lilia Evans has no shareholdings in pharmaceutical companies. Simon Erridge is a junior doctor and Research Director at Curaleaf Clinic. Simon Erridge is a research fellow at Imperial College London. Simon Erridge has no shareholdings in pharmaceutical companies. Madhur Varadpande is a medical student at Imperial College London. Madhur Varadpande has no shareholdings in pharmaceutical companies. Isaac Cowley is a medical student at Imperial College London. Isaac Cowley has no shareholdings in pharmaceutical companies. Arushika Aggarwal is a medical student at Imperial College London. Arushika Aggarwal has no shareholdings in pharmaceutical companies. Evonne Clarke is the Patient Care Director at Curaleaf Clinic. Evonne Clarke has no shareholdings in pharmaceutical companies. Katy McLachlan is the Chief Pharmacist at Curaleaf Clinic. Katy McLachlan has no shareholdings in pharmaceutical companies. Ross Coomber is a consultant orthopedic surgeon at St George's Hospital, London, and Operations Director at Curaleaf Clinic. Ross Coomber has no shareholdings in pharmaceutical companies. James Rucker is a consultant psychiatrist at Curaleaf Clinic. James Rucker is an honorary consultant psychiatrist at The South London & Maudsley NHS Foundation Trust, and an NIHR Clinician Scientist Fellow at the Centre for Affective Disorders at King's College London. James Rucker is funded by a fellowship (CS‐2017‐17‐007) from the National Institute for Health Research (NIHR). The views expressed are those of the author(s) and not necessarily those of the NHS, the NIHR or the Department of Health. James Rucker leads the Psychedelic Trials Group at King's College London. King's College London receives grant funding from COMPASS Pathways PLC to undertake phase 1 and phase 2 trials with psilocybin. COMPASS Pathways PLC has paid for James Rucker to attend trial related meetings and conferences to present the results of research using psilocybin. James Rucker has undertaken paid consultancy work for Beckley PsyTech and Clerkenwell Health. Payments for consultancy work are received and managed by King's College London and James Rucker does not benefit personally. James Rucker has no shareholdings in pharmaceutical companies. Michael Platt is a consultant in pain services at Curaleaf Clinic. Michael Platt has no shareholdings in pharmaceutical companies. Mikael Sodergren is a consultant hepatopancreatobiliary surgeon at Imperial College NHS Trust, London, a senior clinical lecturer at Imperial College London, and the chief medical officer of Curaleaf International. Mikael Sodergren has no shareholdings in pharmaceutical companies.

## Peer Review

The peer review history for this article is available at https://publons.com/publon/10.1002/brb3.70823.

## Supporting information




**Supporting Appendix**: brb370823‐sup‐0001‐AppendixA.pdf

**Supporting Appendix**: brb370823‐sup‐0002‐AppendixB.pdf

**Supporting Appendix**: brb370823‐sup‐0003‐AppendixC.pdf

**Supporting Appendix**: brb370823‐sup‐0004‐AppendixD.pdf

## Data Availability

Data that support the findings of this study are available from the UK Medical Cannabis Registry. Restrictions apply to the availability of these data. Data specifications and applications are available from the corresponding author. All authors contributed to and approved the final article. All work was conducted at Curaleaf Clinic, London, UK.

## References

[brb370823-bib-0001] Abelev, S. , L. N. Warne , M. Benson , M. Hardy , S. Nayee , and J. Barlow . 2022. “Medicinal Cannabis for the Treatment of Chronic Refractory Pain: An Investigation of the Adverse Event Profile and Health‐Related Quality of Life Impact of an Oral Formulation.” Medical Cannabis and Cannabinoids 5, no. 1: 20–31. 10.1159/000521492. 35950052 PMC9235063

[brb370823-bib-0002] Anand, U. , B. Jones , Y. Korchev , et al. 2020. “CBD Effects on TRPV1 Signaling Pathways in Cultured DRG Neurons.” Journal of Pain Research 13: 2269–2278. 10.2147/JPR.S258433. 32982390 PMC7494392

[brb370823-bib-0003] Anand, U. , W. R. Otto , D. Sanchez‐Herrera , et al. 2008. “Cannabinoid Receptor CB2 Localisation and Agonist‐Mediated Inhibition of Capsaicin Responses in Human Sensory Neurons.” Pain 138, no. 3: 667–680. 10.1016/J.PAIN.2008.06.007. 18692962

[brb370823-bib-0004] Andreas, G. , B. CH , T.‐S. Lynne , et al. 2012. Complex Regional Pain Syndrome: UK Guidelines for Diagnosis, Referral and Management in Primary and Secondary Care. Royal College of Physicians.

[brb370823-bib-0005] Bapir, L. , S. Erridge , M. Nicholas , et al. 2023. “Comparing the Effects of Medical Cannabis for Chronic Pain Patients With and Without Co‐Morbid Anxiety: A Cohort Study.” Expert Review of Neurotherapeutics 23, no. 3: 281–295. 10.1080/14737175.2023.2181696. 36803620

[brb370823-bib-0006] BMJ Best Practice . n.d. “Complex Regional Pain Syndrome—Treatment Algorithm.” Accessed April 23, 2024. https://bestpractice.bmj.com/topics/en‐gb/594/treatment‐algorithm.

[brb370823-bib-0007] Busse, J. W. , L. Wang , M. Kamaleldin , et al. 2018. “Opioids for Chronic Noncancer Pain: A Systematic Review and Meta‐Analysis.” JAMA 320, no. 23: 2448–2460. 10.1001/JAMA.2018.18472. 30561481 PMC6583638

[brb370823-bib-0008] Cahill, S. P. , S. E. Lunn , P. Diaz , and J. E Page . 2021. “Evaluation of Patient Reported Safety and Efficacy of Cannabis From a Survey of Medical Cannabis Patients in Canada.” Frontiers in Public Health 9: 626853. 10.3389/FPUBH.2021.626853. 34095048 PMC8172603

[brb370823-bib-0009] Charlson, M. E. , P. Pompei , K. L. Ales , and C. R MacKenzie . 1987. “A New Method of Classifying Prognostic Comorbidity in Longitudinal Studies: Development and Validation.” Journal of Chronic Diseases 40, no. 5: 373–383. 10.1016/0021-9681(87)90171-8. 3558716

[brb370823-bib-0010] Childs, E. , J. A. Lutz , and H. de Wit . 2017. “Dose‐Related Effects of Delta‐9‐THC on Emotional Responses to Acute Psychosocial Stress.” Drug and Alcohol Dependence 177: 136–144. 10.1016/J.DRUGALCDEP.2017.03.030. 28599212 PMC6349031

[brb370823-bib-0011] Cohen, S. P. , L. Vase , and W. M Hooten . 2021. “Chronic Pain: An Update on Burden, Best Practices, and New Advances.” Lancet 397, no. 10289: 2082–2097. 10.1016/S0140-6736(21)00393-7. 34062143

[brb370823-bib-0012] Committee for Medicinal Products for Human Use (CHMP) . 2010. “Guideline on Missing Data in Confirmatory Clinical Trials.” Published July 2. https://www.ema.europa.eu/en/documents/scientific‐guideline/guideline‐missing‐data‐confirmatory‐clinical‐trials_en.pdf.

[brb370823-bib-0013] Corroon, J. M. , L. K. Mischley , and M Sexton . 2017. “Cannabis as a Substitute for Prescription Drugs—A Cross‐Sectional Study.” Journal of Pain Research 10: 989–998. 10.2147/JPR.S134330. 28496355 PMC5422566

[brb370823-bib-0014] Dansie, E. J. , and D. C Turk . 2013. “Assessment of Patients With Chronic Pain.” British Journal of Anaesthesia 111, no. 1: 19–25. 10.1093/BJA/AET124. 23794641 PMC3841375

[brb370823-bib-0015] Degenhardt, L. , J. Grebely , J. Stone , et al. 2019. “Global Patterns of Opioid Use and Dependence: Harms to Populations, Interventions, and Future Action.” Lancet 394, no. 10208: 1560–1579. 10.1016/S0140-6736(19)32229-9. 31657732 PMC7068135

[brb370823-bib-0016] de Mos, M. , A. G. J. de Bruijn , F. Huygen , J. P. Dieleman , B. H. C. Stricker , and M. Sturkenboom . 2007. “The Incidence of Complex Regional Pain Syndrome: A Population‐Based Study.” Pain 129, no. 1–2: 12–20. 10.1016/J.PAIN.2006.09.008. 17084977

[brb370823-bib-0017] Deutsch, D. G. 2016. “A Personal Retrospective: Elevating Anandamide (AEA) by Targeting Fatty Acid Amide Hydrolase (FAAH) and the Fatty Acid Binding Proteins (FABPs).” Frontiers in Pharmacology 7: 370. 10.3389/FPHAR.2016.00370. 27790143 PMC5062061

[brb370823-bib-0018] Dey, S. , K. B. Guthmiller , and M Varacallo . 2023. Complex Regional Pain Syndrome. StatPearls Publishing. https://www.ncbi.nlm.nih.gov/books/NBK430719/. 28613470

[brb370823-bib-0019] Di Stefano, G. , A. Di Lionardo , G. Di Pietro , G. Cruccu , and A Truini . 2021. “Pharmacotherapeutic Options for Managing Neuropathic Pain: A Systematic Review and Meta‐Analysis.” Pain Research and Management 2021, no. 1: 6656863. 10.1155/2021/6656863. 33986899 PMC8093054

[brb370823-bib-0020] Dworkin, R. H. , D. C. Turk , K. W. Wyrwich , et al. 2008. “Interpreting the Clinical Importance of Treatment Outcomes in Chronic Pain Clinical Trials: IMMPACT Recommendations.” Journal of Pain 9, no. 2: 105–121. 10.1016/J.JPAIN.2007.09.005. 18055266

[brb370823-bib-0021] Elsamadicy, A. A. , S. Yang , A. R. Sergesketter , et al. 2018. “Prevalence and Cost Analysis of Complex Regional Pain Syndrome (CRPS): A Role for Neuromodulation.” Neuromodulation 21, no. 5: 423–430. 10.1111/NER.12691. 28961359 PMC5876058

[brb370823-bib-0022] Erridge, S. , O. Leung , C. Holvey , et al. 2023. “An Observational Study of Clinical Outcome Measures in Patients Treated With Cannabis‐Based Medicinal Products on the UK Medical Cannabis Registry.” Neuropsychopharmacology Reports 43, no. 4: 616–632. 10.1002/NPR2.12403. 38057993 PMC10739137

[brb370823-bib-0023] Erridge, S. , O. Salazar , M. Kawka , et al. 2021. “An Initial Analysis of the UK Medical Cannabis Registry: Outcomes Analysis of First 129 Patients.” Neuropsychopharmacology Reports 41, no. 3: 362–370. 10.1002/npr2.12183. 33988306 PMC8411316

[brb370823-bib-0024] Erridge, S. , L. Troup , and M. H. Sodergren . 2024. “Illicit Cannabis Use to Self‐Treat Chronic Health Conditions: A Cross‐Sectional Study From the United Kingdom.” JMIR Public Health and Surveillance 10: e57595. 10.2196/preprints.57595. 39149844 PMC11337234

[brb370823-bib-0025] Ferguson, L. , and J Scheman . 2009. “Patient Global Impression of Change Scores Within the Context of a Chronic Pain Rehabilitation Program.” Journal of Pain 10, no. 4: S73. 10.1016/j.jpain.2009.01.258.

[brb370823-bib-0026] Ferraro, M. C. , A. G. Cashin , B. M. Wand , et al. 2023. “Interventions for Treating Pain and Disability in Adults With Complex Regional Pain Syndrome–An Overview of Systematic Reviews.” Cochrane Database of Systematic Reviews 2023, no. 6: CD009416. 10.1002/14651858.CD009416.PUB3. PMC10259367 37306570

[brb370823-bib-0027] Ferraro, M. C. , N. E. O'Connell , C. Sommer , et al. 2024. “Complex Regional Pain Syndrome: Advances in Epidemiology, Pathophysiology, Diagnosis, and Treatment.” Lancet Neurology 23, no. 5: 522–533. 10.1016/S1474-4422(24)00076-0. 38631768

[brb370823-bib-0028] Fine, M. 2013. “Quantifying the Impact of NSAID‐Associated Adverse Events.” American Journal of Managed Care 19, no. S14: S267–S272. https://www.ajmc.com/view/a467_nov13_fine_s267. 24494609

[brb370823-bib-0029] Finnmann Munk, A. S. , K. K. Petersen , S. Bødtker , et al. 2022. “Long‐Term Biopsychosocial Issues and Health‐Related Quality of Life in Young Adolescents and Adults Treated for Childhood Complex Regional Pain Syndrome, Type 1.” Scandinavian Journal of Pain 22, no. 3: 473–482. 10.1515/SJPAIN-2021-0217/MACHINEREADABLECITATION/RIS. 35639860

[brb370823-bib-0030] González‐Ramírez, R. , Y. Chen , W. B. Liedtke , and S. L Morales‐Lázaro . 2017. “TRP Channels and Pain.” Neurobiology of TRP Channels 1: 125–148. 10.4324/9781315152837-8. 29356491

[brb370823-bib-0031] Goodman, C. W. , and A. S Brett . 2017. “Gabapentin and Pregabalin for Pain—Is Increased Prescribing a Cause for Concern?” New England Journal of Medicine 377, no. 5: 411–414. 10.1056/NEJMp1704633. 28767350

[brb370823-bib-0032] Haefeli, M. , and A Elfering . 2006. “Pain Assessment.” European Spine Journal 15, no. S1: S17. 10.1007/S00586-005-1044-X. 16320034 PMC3454549

[brb370823-bib-0033] Hernandez, G. , O. Garin , A. L. Dima , et al. 2019. “EuroQol (EQ‐5D‐5L) Validity in Assessing the Quality of Life in Adults With Asthma: Cross‐Sectional Study.” Journal of Medical Internet Research 21, no. 1: e10178. 10.2196/10178. 30672744 PMC6364208

[brb370823-bib-0034] Hill, K. P. , M. D. Palastro , B. Johnson , and J. W Ditre . 2017. “Cannabis and Pain: A Clinical Review.” Cannabis and Cannabinoid Research 2, no. 1: 96. 10.1089/CAN.2017.0017. 28861509 PMC5549367

[brb370823-bib-0035] Hsieh, G. C. , M. Pai , P. Chandran , et al. 2011. “Central and Peripheral Sites of Action for CB2 Receptor Mediated Analgesic Activity in Chronic Inflammatory and Neuropathic Pain Models in Rats.” British Journal of Pharmacology 162, no. 2: 428. 10.1111/J.1476-5381.2010.01046.X. 20880025 PMC3031063

[brb370823-bib-0036] Hylands‐White, N. , R. V. Duarte , and J. H Raphael . 2017. “An Overview of Treatment Approaches for Chronic Pain Management.” Rheumatology International 37, no. 1: 29–42. 10.1007/s00296-016-3481-8. 27107994

[brb370823-bib-0037] IBM Corp . 2023. “IBM SPSS Statistics for Windows, Version 29.0.2.0.” https://www.ibm.com/products/spss‐statistics.

[brb370823-bib-0038] Ibrahim, M. M. , F. Porreca , J. Lai , et al. 2005. “CB2 Cannabinoid Receptor Activation Produces Antinociception by Stimulating Peripheral Release of Endogenous Opioids.” Proceedings of the National Academy of Sciences of the United States of America 102, no. 8: 3093–3098. 10.1073/pnas.0409888102. 15705714 PMC549497

[brb370823-bib-0039] International Labour Organization . n.d. “International Standard Classification of Occupations.” Accessed May 23, 2024. https://ilostat.ilo.org/methods/concepts‐and‐definitions/classification‐occupation/.

[brb370823-bib-0040] Jeddi, H. M. , J. W. Busse , B. Sadeghirad , et al. 2024. “Cannabis for Medical Use Versus Opioids for Chronic Non‐Cancer Pain: A Systematic Review and Network Meta‐Analysis of Randomised Clinical Trials.” BMJ Open 14, no. 1: e068182. 10.1136/BMJOPEN-2022-068182. PMC10773353 38171632

[brb370823-bib-0041] Johns, A. 2001. “Psychiatric Effects of Cannabis.” British Journal of Psychiatry 178, no. 2: 116–122. 10.1192/BJP.178.2.116. 11157424

[brb370823-bib-0042] Katz, J. , and R Melzack . n.d. “The McGill Pain Questionnaire.” Accessed April 25, 2024. https://core.ac.uk/download/pdf/10988388.pdf.

[brb370823-bib-0043] Kelley, M. D. , M. Obaid , E. M. Miller , M. Bowie , and Z. S Heeter . 2024. “Observational Analysis of the Influence of Medical Marijuana Use on Quality of Life in Patients.” Medical Cannabis and Cannabinoids 7, no. 1: 44–50. 10.1159/000536591. 38500669 PMC10948168

[brb370823-bib-0044] Laprairie, R. B. , A. M. Bagher , M. E. M. Kelly , and E. M Denovan‐Wright . 2015. “Cannabidiol Is a Negative Allosteric Modulator of the Cannabinoid CB1 Receptor.” British Journal of Pharmacology 172, no. 20: 4790. 10.1111/BPH.13250. 26218440 PMC4621983

[brb370823-bib-0045] Lim, G. , B. Sung , R. R. Ji , and J Mao . 2003. “Upregulation of Spinal Cannabinoid‐1‐Receptors Following Nerve Injury Enhances the Effects of Win 55,212‐2 on Neuropathic Pain Behaviors in Rats.” Pain 105, no. 1–2: 275–283. 10.1016/S0304-3959(03)00242-2. 14499445

[brb370823-bib-0046] Linge, R. , L. Jiménez‐Sánchez , L. Campa , et al. 2016. “Cannabidiol Induces Rapid‐Acting Antidepressant‐Like Effects and Enhances Cortical 5‐HT/Glutamate Neurotransmission: Role of 5‐HT1A Receptors.” Neuropharmacology 103: 16–26. 10.1016/J.NEUROPHARM.2015.12.017. 26711860

[brb370823-bib-0047] Liu‐Seifert, H. , S. Zhang , D. D'Souza , and V Skljarevski . 2010. “A Closer Look at the Baseline‐Observation‐Carried‐Forward (BOCF).” Patient Preference and Adherence 4: 11. 10.2147/PPA.S8135. 20165594 PMC2819899

[brb370823-bib-0048] Llewellyn, A. , M. J. Sweeting , and C McCabe . 2019. “Understanding the Biopsychosocial Impacts of Living With Complex Regional Pain Syndrome.” https://uwe‐repository.worktribe.com/index.php/output/851697/understanding‐the‐biopsychosocial‐impacts‐of‐living‐with‐complex‐regional‐pain‐syndrome.

[brb370823-bib-0049] Lucas, C. J. , P. Galettis , and J Schneider . 2018. “The Pharmacokinetics and the Pharmacodynamics of Cannabinoids.” British Journal of Clinical Pharmacology 84, no. 11: 2477–2482. 10.1111/BCP.13710. 30001569 PMC6177698

[brb370823-bib-0050] MacHado, G. C. , C. Abdel‐Shaheed , M. Underwood , and R. O Day . 2021. “Non‐Steroidal Anti‐inflammatory Drugs (NSAIDs) for Musculoskeletal Pain.” Bmj 372: n104. 10.1136/BMJ.N104. 33514562

[brb370823-bib-0051] Maldonado, R. , J. E. Baños , and D Cabañero . 2016. “The Endocannabinoid System and Neuropathic Pain.” Pain 157: S23–S32. 10.1097/J.PAIN.0000000000000428. 26785153

[brb370823-bib-0052] May, M. , D. U. Junghaenel , M. Ono , A. A. Stone , and S Schneider . 2018. “Ecological Momentary Assessment Methodology in Chronic Pain Research: A Systematic Review.” Journal of Pain 19, no. 7: 699–716. 10.1016/J.JPAIN.2018.01.006. 29371113 PMC6026050

[brb370823-bib-0053] Mcneilage, A. G. , S. Nielsen , B. Murnion , and C Ashton‐James . 2023. “Experiences of Misuse and Symptoms of Dependence Among People Who Use Gabapentinoids: A Qualitative Systematic Review Protocol.” BMJ Open 13: 73770. 10.1136/bmjopen-2023-073770. PMC10546131 37775298

[brb370823-bib-0054] MD Anderson Cancer Center . n.d. “Brief Pain Inventory.” Accessed April 25, 2024. https://www.mdanderson.org/research/departments‐labs‐institutes/departments‐divisions/symptom‐research/symptom‐assessment‐tools/brief‐pain‐inventory.html.

[brb370823-bib-0055] Meints, S. M. , and R. R Edwards . 2018. “Evaluating Psychosocial Contributions to Chronic Pain Outcomes.” Progress in Neuro‐Psychopharmacology and Biological Psychiatry 87: 168–182. 10.1016/J.PNPBP.2018.01.017. 29408484 PMC6067990

[brb370823-bib-0056] Moore, R. A. , C. C. Chi , P. J. Wiffen , S. Derry , and A. S. C Rice . 2015. “Oral Nonsteroidal Anti‐Inflammatory Drugs for Neuropathic Pain.” Cochrane Database of Systematic Reviews 2015, no. 10: CD010902. 10.1002/14651858.CD010902.PUB2. 26436601 PMC6481590

[brb370823-bib-0057] Moore, R. A. , E. Fisher , D. P. Finn , et al. 2021. “Cannabinoids, Cannabis, and Cannabis‐Based Medicines for Pain Management: An Overview of Systematic Reviews.” Pain 162: S67–S79. 10.1097/J.PAIN.0000000000001941. 32804833

[brb370823-bib-0058] Mücke, M. , T. Phillips , L. Radbruch , F. Petzke , and W Häuser . 2018. “Cannabis‐Based Medicines for Chronic Neuropathic Pain in Adults.” Cochrane Database of Systematic Reviews 2018, no. 3: CD012182. 10.1002/14651858.CD012182.pub2. PMC6494210 29513392

[brb370823-bib-0059] Munro, S. , K. L. Thomas , and M Abu‐Shaar . 1993. “Molecular Characterization of a Peripheral Receptor for Cannabinoids.” Nature 365, no. 6441: 61–65. 10.1038/365061a0. 7689702

[brb370823-bib-0060] Naleschinski, D. , and R Baron . 2010. “Complex Regional Pain Syndrome Type I: Neuropathic or Not?” Current Pain and Headache Reports 14, no. 3: 196–202. 10.1007/S11916-010-0115-9. 20461475

[brb370823-bib-0061] National Cancer Institute . 2009 “Lead Organizations: NCI Network Trial Development and Conduct.” Accessed May 1, 2024. https://ctep.cancer.gov/protocolDevelopment/electronic_applications/ctc.htm.

[brb370823-bib-0062] NHS . 2022 “Complex Regional Pain Syndrome.” Accessed April 23, 2024. https://www.nhs.uk/conditions/complex‐regional‐pain‐syndrome/.

[brb370823-bib-0063] Nutt, D. J. , L. D. Phillips , M. P. Barnes , et al. 2022. “A Multicriteria Decision Analysis Comparing Pharmacotherapy for Chronic Neuropathic Pain, Including Cannabinoids and Cannabis‐Based Medical Products.” Cannabis and Cannabinoid Research 7, no. 4: 482. 10.1089/CAN.2020.0129. 33998895 PMC9418467

[brb370823-bib-0064] Olsson, F. , S. Erridge , J. Tait , et al. 2023. “An Observational Study of Safety and Clinical Outcome Measures Across Patient Groups in the United Kingdom Medical Cannabis Registry.” Expert Review of Clinical Pharmacology 16, no. 3: 257–266. 10.1080/17512433.2023.2183841. 36848456

[brb370823-bib-0065] Pertwee, R. G. 2008. “The Diverse CB1 and CB2 Receptor Pharmacology of Three Plant Cannabinoids: Δ9‐Tetrahydrocannabinol, Cannabidiol and Δ9‐Tetrahydrocannabivarin.” British Journal of Pharmacology 153, no. 2: 199–215. 10.1038/SJ.BJP.0707442. 17828291 PMC2219532

[brb370823-bib-0066] Quan, H. , B. Li , C. M. Couris , et al. 2011. “Updating and Validating the Charlson Comorbidity Index and Score for Risk Adjustment in Hospital Discharge Abstracts Using Data From 6 Countries.” American Journal of Epidemiology 173, no. 6: 676–682. 10.1093/AJE/KWQ433. 21330339

[brb370823-bib-0067] Rahn, E. J. , and A. G Hohmann . 2009. “Cannabinoids as Pharmacotherapies for Neuropathic Pain: From the Bench to the Bedside.” Neurotherapeutics 6, no. 4: 713–737. 10.1016/J.NURT.2009.08.002. 19789075 PMC2755639

[brb370823-bib-0068] Rapin, L. , R. Gamaoun , C. El Hage , M. F. Arboleda , and E Prosk . 2021. “Cannabidiol Use and Effectiveness: Real‐World Evidence From a Canadian Medical Cannabis Clinic.” Journal of Cannabis Research 3, no. 1: 1–10. 10.1186/S42238-021-00078-W. 34162446 PMC8223341

[brb370823-bib-0069] Rog, D. J. , T. J. Nurmikko , T. Friede , and C. A Young . 2005. “Randomized, Controlled Trial of Cannabis‐Based Medicine in Central Pain in Multiple Sclerosis.” Neurology 65, no. 6: 812–819. 10.1212/01.WNL.0000176753.45410.8B/SUPPL_FILE/E3.DOC. 16186518

[brb370823-bib-0070] Ryberg, E. , N. Larsson , S. Sjögren , et al. 2007. “The Orphan Receptor GPR55 Is a Novel Cannabinoid Receptor.” British Journal of Pharmacology 152, no. 7: 1092–1101. 10.1038/SJ.BJP.0707460. 17876302 PMC2095107

[brb370823-bib-0071] Shirvalkar, P. , J. Prosky , G. Chin , et al. 2023. “First‐In‐Human Prediction of Chronic Pain state Using Intracranial Neural Biomarkers.” Nature Neuroscience 26, no. 6: 1090–1099. 10.1038/s41593-023-01338-z. 37217725 PMC10330878

[brb370823-bib-0072] Snyder, E. , B. Cai , C. DeMuro , M. F. Morrison , and W Ball . 2018. “A New Single‐Item Sleep Quality Scale: Results of Psychometric Evaluation in Patients With Chronic Primary Insomnia and Depression.” Journal of Clinical Sleep Medicine 14, no. 11: 1849–1857. 10.5664/jcsm.7478. 30373688 PMC6223557

[brb370823-bib-0073] Spitzer, R. L. , K. Kroenke , J. B. W. Williams , and B Löwe . 2006. “A Brief Measure for Assessing Generalized Anxiety Disorder: The GAD‐7.” Archives of Internal Medicine 166, no. 10: 1092–1097. 10.1001/ARCHINTE.166.10.1092. 16717171

[brb370823-bib-0074] Stella, B. , F. Baratta , C. Della Pepa , S. Arpicco , D. Gastaldi , and F Dosio . 2021. “Cannabinoid Formulations and Delivery Systems: Current and Future Options to Treat Pain.” Drugs 81, no. 13: 1513–1557. 10.1007/S40265-021-01579-X. 34480749 PMC8417625

[brb370823-bib-0075] Tait, J. , S. Erridge , C. Holvey , et al. 2023. “Clinical Outcome Data of Chronic Pain Patients Treated With Cannabis‐Based Oils and Dried Flower From the UK Medical Cannabis Registry.” Expert Review of Neurotherapeutics 23, no. 4: 413–423. 10.1080/14737175.2023.2195551. 37021592

[brb370823-bib-0076] Tait, J. , S. Erridge , and M. H Sodergren . 2023. “UK Medical Cannabis Registry: A Patient Evaluation.” Journal of Pain & Palliative Care Pharmacotherapy 37, no. 2: 170–177. 10.1080/15360288.2023.2174633. 36762986

[brb370823-bib-0077] Tait, M. A. , D. S. J. Costa , R. Campbell , et al. 2023. “Health‐Related Quality of Life in Patients Accessing Medicinal Cannabis in Australia: The QUEST Initiative Results of a 3‐Month Follow‐Up Observational Study.” PLoS ONE 18: e0290549. 10.1371/journal.pone.0290549. 37672515 PMC10482296

[brb370823-bib-0078] Takakuwa, K. M. , J. Y. Hergenrather , F. S. Shofer , and R. M Schears . 2020. “The Impact of Medical Cannabis on Intermittent and Chronic Opioid Users With Back Pain: How Cannabis Diminished Prescription Opioid Usage.” Cannabis and Cannabinoid Research 5, no. 3: 263–270. 10.1089/CAN.2019.0039. 32923663 PMC7480723

[brb370823-bib-0079] Thoma, P. , N. Drämel , M. Grothe , M. Lotze , R. Fleischmann , and S Strauss . 2022. “Impaired Pain Processing at a Brainstem Level Is Involved in Maladaptive Neuroplasticity in Patients With Chronic Complex Regional Pain Syndrome.” International Journal of Molecular Sciences 23, no. 23: 15368. 10.3390/ijms232315368. 36499694 PMC9740440

[brb370823-bib-0080] Toussaint, A. , P. Hüsing , A. Gumz , et al. 2020. “Sensitivity to Change and Minimal Clinically Important Difference of the 7‐Item Generalized Anxiety Disorder Questionnaire (GAD‐7).” Journal of Affective Disorders 265: 395–401. 10.1016/J.JAD.2020.01.032. 32090765

[brb370823-bib-0081] Trotti, A. , A. D. Colevas , A. Setser , and E Basch . 2016. “Patient‐Reported Outcomes and the Evolution of Adverse Event Reporting in Oncology.” Journal of Clinical Oncology 25, no. 32: 5121–5127. 10.1200/JCO.2007.12.4784. 17991931

[brb370823-bib-0082] Turk, D. C. , R. B. Fillingim , R. Ohrbach , and K. V Patel . 2016. “Assessment of Psychosocial and Functional Impact of Chronic Pain.” Journal of Pain 17, no. 9: T21–T49. 10.1016/J.JPAIN.2016.02.006. 27586830

[brb370823-bib-0083] Vigil, J. M. , S. S. Stith , I. M. Adams , and A. P Reeve . 2017. “Associations Between Medical Cannabis and Prescription Opioid Use in Chronic Pain Patients: A Preliminary Cohort Study.” PLoS ONE 12, no. 11: e0187795. 10.1371/JOURNAL.PONE.0187795. 29145417 PMC5690609

[brb370823-bib-0084] Vitali, D. , T. Olugbade , C. Eccleston , E. Keogh , N. Bianchi‐Berthouze , and A. C De C Williams . 2024. “Sensing Behavior Change in Chronic Pain: A Scoping Review of Sensor Technology for Use in Daily Life.” Pain 165, no. 6: 1348–1360. 10.1097/J.PAIN.0000000000003134. 38258888

[brb370823-bib-0085] Von Elm, E. , D. G. Altman , M. Egger , S. J. Pocock , P. C. Gøtzsche , and J. P Vandenbrouckef . 2007. “The Strengthening the Reporting of Observational Studies in Epidemiology (STROBE) Statement: Guidelines for Reporting Observational Studies.” Bulletin of the World Health Organization 85, no. 11: 867–872. 10.2471/BLT.07.045120. 18038077 PMC2636253

[brb370823-bib-0086] Vučkovic, S. , D. Srebro , K. S. Vujovic , Č. Vučetic , and M Prostran . 2018. “Cannabinoids and Pain: New Insights From Old Molecules.” Frontiers in Pharmacology 9: 1259. 10.3389/FPHAR.2018.01259. 30542280 PMC6277878

[brb370823-bib-0087] Wang, L. , P. J. Hong , C. May , et al. 2021. “Medical Cannabis or Cannabinoids for Chronic Non‐Cancer and Cancer Related Pain: A Systematic Review and Meta‐Analysis of Randomised Clinical Trials.” Bmj 374: n1034. 10.1136/BMJ.N1034. 34497047

[brb370823-bib-0088] Ware, M. A. , H. Adams , and G. W Guy . 2005. “The Medicinal Use of Cannabis in the UK: Results of a Nationwide Survey.” International Journal of Clinical Practice 59, no. 3: 291–295. 10.1111/J.1742-1241.2004.00271.X. 15857325

[brb370823-bib-0089] Ware, M. A. , T. Wang , S. Shapiro , et al. 2010. “Smoked Cannabis for Chronic Neuropathic Pain: A Randomized Controlled Trial.” Cmaj 182, no. 14: E694–E701. 10.1503/CMAJ.091414. 20805210 PMC2950205

[brb370823-bib-0090] Wilsey, B. , T. Marcotte , R. Deutsch , B. Gouaux , S. Sakai , and H Donaghe . 2013. “Low‐Dose Vaporized Cannabis Significantly Improves Neuropathic Pain.” Journal of Pain 14, no. 2: 136–148. 10.1016/j.jpain.2012.10.009. 23237736 PMC3566631

[brb370823-bib-0091] Wilsey, B. , T. Marcotte , A. Tsodikov , et al. 2008. “A Randomized, Placebo‐Controlled, Crossover Trial of Cannabis Cigarettes in Neuropathic Pain.” Journal of Pain 9, no. 6: 506–521. 10.1016/J.JPAIN.2007.12.010. 18403272 PMC4968043

[brb370823-bib-0092] Zhang, J. , C. Hoffert , H. K. Vu , T. Groblewski , S. Ahmad , and D O'Donnell . 2003. “Induction of CB2 Receptor Expression in the Rat Spinal Cord of Neuropathic but Not Inflammatory Chronic Pain Models.” European Journal of Neuroscience 17, no. 12: 2750–2754. 10.1046/J.1460-9568.2003.02704.X. 12823482

