# Supplementary material for: UK Medical Cannabis Registry: A Clinical Outcomes Analysis for Complex Regional Pain Syndrome
Source: Brain Behav. 2025 Sep 2;15(9):e70823. doi: 10.1002/brb3.70823 (PMC12405601; doi:10.1002/brb3.70823)
Supplement: Supplementary file 1 — Supporting Appendix: brb370823‐sup‐0001‐AppendixA.pdf [file BRB3-15-e70823-s004.pdf]

<u>Appendix A:</u> Table of study participants' geographical distribution across the United Kingdom (n=64).

**Region of the UK**	***n* (%)**
East Midlands	2 (3.10)
East of England	5 (7.80)
Isle of Man	1 (1.60)
London	1 (1.60)
North East England	3 (4.70)
North West England	5 (7.80)
Northern Ireland	1 (1.60)
Scotland	13 (20.30)
South East England	10 (15.60)
South West England	6 (9.40)
Wales	4 (6.30)
West Midlands	10 (15.60)
Yorkshire and the Humber	3 (4.70)

UK = United Kingdom.
