# Supplementary material for: UK Medical Cannabis Registry: A Clinical Outcomes Analysis for Complex Regional Pain Syndrome
Source: Brain Behav. 2025 Sep 2;15(9):e70823. doi: 10.1002/brb3.70823 (PMC12405601; doi:10.1002/brb3.70823)
Supplement: Supplementary file 2 — Supporting Appendix: brb370823‐sup‐0002‐AppendixB.pdf [file BRB3-15-e70823-s003.pdf]

<u>Appendix B:</u> **Repeated measures ANOVA of patient-reported outcome measures at baseline, 1 month, 3 month and 6 months**. Values for the mean and standard deviation were calculated from raw data in IBM Statistical Package for Social Sciences (SPSS) version 29. BPI Pain Severity & Interference, Pain VAS, EQ-5D-5L, GAD-7, PGIC and SQS. (n=64). SF-MPQ-2 Neuropathic pain, affective descriptors, continuous pain, intermittent pain and overall score (n=63). P-values shown; (***=p<0.001, **=p<0.010, *=p<0.050)**.** Green shading = p<0.050 Red shading = p≥0.050.

PROMs	Baseline	1 month	3 months	6 months	p-value
**BPI Pain Severity**	6.69 ± 1.42	5.85 ± 1.73	5.91 ± 1.82	6.05 ± 1.72	<0.001***
**BPI Pain Interference**	7.90 ±1.62	6.73 ± 2.19	6.56 ± 2.33	6.87 ± 2.30	<0.001***
**SF-MPQ-2 Neuropathic Pain**	5.84 ±2.29	5.07 ± 2.32	4.91 ± 2.34	4.91 ± 2.23	<0.001***
**SF-MPQ-2 Affective Descriptors**	6.00 ±2.38	4.92 ± 2.66	4.66 ± 2.62	4.86 ± 2.53	<0.001***
**SF-MPQ-2 Continuous Pain**	6.55 ±2.04	5.82 ± 2.47	5.76 ± 2.25	5.75 ± 2.27	0.004**
**SF-MPQ-2 Intermittent Pain**	6.30 ±2.04	5.69 ± 2.37	5.13 ± 2.44	5.51 ± 2.29	<0.001***
**SF-MPQ-2 Overall Score**	6.17 ±1.79	5.37 ± 2.07	5.11 ± 2.07	5.26 ± 2.00	<0.001***
**Pain VAS**	7.86 ±1.61	6.94 ± 2.09	6.86 ± 2.30	6.79 ± 2.11	<0.001***
**EQ-5D-5L Index Value**	0.13 ± 0.30	0.34 ± 0.30	0.31 ± 0.35	0.30 ± 0.33	<0.001***
**EQ-5D-5L Mobility**	3.45±1.04	3.19±1.07	3.28±1.16	3.19±1.1	0.044*
**EQ-5D-5L Self-Care**	2.41±1.12	2.36±1.03	2.42±1.11	2.37±1.09	0.866
**EQ-5D-5L Usual Activities**	3.58±1.01	3.12±1.12	3.30±1.06	3.28±1.00	0.001***
**EQ-5D-5L Pain and Discomfort**	4.30±0.73	3.52±0.96	3.53±1.07	3.72±0.95	<0.001***
**EQ-5D-5L Anxiety and Depression**	2.84±1.30	2.30±1.05	2.42±1.22	2.44±1.22	<0.001***
**GAD-7**	9.59±6.47	6.31±4.84	7.56±6.01	7.42±5.70	<0.001***
**PGIC**		4.91±1.47	5.05±1.46	5.11±1.56	0.424
**SQS**	3.17±2.01	4.41±2.63	4.56±2.56	4.30±2.62	<0.001***

BPI = Brief Pain Inventory, SF-MPQ-2 = Short Form McGill Pain Questionnaire-2, Pain VAS = Pain Visual Analogue Scale, EQ-5D-5L = European Quality of Life 5 Dimension – 5 Level, GAD-7 = Generalised-Anxiety Disorder, PGIC = Patient Global Impression of Change, SQS = Sleep Quality Scale.
