# Supplementary material for: UK Medical Cannabis Registry: A Clinical Outcomes Analysis for Complex Regional Pain Syndrome
Source: Brain Behav. 2025 Sep 2;15(9):e70823. doi: 10.1002/brb3.70823 (PMC12405601; doi:10.1002/brb3.70823)
Supplement: Supplementary file 3 — Supporting Appendix: brb370823‐sup‐0003‐AppendixC.pdf [file BRB3-15-e70823-s002.pdf]

<u>Appendix C:</u> Univariate regression for pain-specific patient-reported outcome measures. Odds ratios and 95% confidence intervals with corresponding p-values were calculated in IBM Statistical Package for Social Sciences (SPSS) version 29. P-values shown; (***=p<0.001, **=p<0.010, *=p<0.050). Green shading = p<0.050

BPI Pain Severity				
**Variable**		**n**	**OR (95% CI)**	**p-value**
**Age (years)**	18-40	29	1	
	40+	35	2.84 (0.87-9.25)	0.084
**BMI (kg/m²)**	≤25	25	1	
	25-30	17	0.97 (0.23-4.15)	0.972
	30+	20	1.71 (0.47-6.25)	0.421
**Gender**	Female	31	1	
	Male	33	0.49 (0.16-1.50)	0.208
**Cannabis Status**	Never Used	22	1	
	Current or Ex-User	42	3.52 (0.89-13.87)	0.072
**CBD Dose**	≤Median Dose of Cohort (≤20.00mg/day)	26	1	
	≥Median Dose of Cohort (≥20.00mg/day)	38	1.11 (0.36-3.37)	0.860
**THC Dose**	≤Median Dose of Cohort (≤117.86mg/day)	32	1	
	≥Median Dose of Cohort (≥117.86mg/day)	32	2.60 (0.83-8.13)	0.101
**Route of CBMP Administration**	Oils	18	1	
	Dried Flower or Both	46	0.41 (0.10-1.65)	0.211

Pain VAS				
**Variable**		**n**	**OR (95% CI)**	**p-value**
**Age (years)**	18-40	29	1	
	40+	34	1.75 (0.62-4.95)	0.288
**BMI (kg/m²)**	≤25	25	1	
	25-30	16	1.07 (0.29-3.92)	0.963
	30+	20	1.19 (0.35-3.98)	0.783
**Gender**	Female	31	1	
	Male	32	0.95 (0.34-2.63)	0.921
**Cannabis Status**	Never Used	21	1	
	Current or Ex-User	42	6.00 (1.53-23.46)	0.010*
**CBD Dose**	≤Median Dose of Cohort (≤20.00mg/day)	26	1	
	≥Median Dose of Cohort (≥20.00mg/day)	37	0.74 (0.26-2.07)	0.564
**THC Dose**	≤Median Dose of Cohort (≤117.86mg/day)	31	1	
	≥Median Dose of Cohort (≥117.86mg/day)	32	1.24 (0.45-3.45)	0.675
**Route of CBMP Administration**	Oils	18	1	
	Dried Flower or Both	45	1.90 (0.58-6.24)	0.29

SF-MPQ-2 Overall Score				
Variable		n	OR (95% CI)	p-value
Age (years)	18-40	29	1	
	40+	34	1.29 (0.47-3.55)	0.619
BMI (kg/m²)	≤25	25	1	
	25-30	16	0.90 (0.25-3.27)	0.873
	30+	20	1.23 (0.37-4.03)	0.736
Gender	Female	31	1	
	Male	32	0.56 (0.20-1.54)	0.261
Cannabis Status	Never Used	21	1	
	Current or Ex-User	42	3.20 (0.99-10.33)	0.052
CBD Dose	≤Median Dose of Cohort (≤20.00mg/day)	26	1	
	≥Median Dose of Cohort (≥20.00mg/day)	37	1.61 (0.57-4.52)	0.370
THC Dose	≤Median Dose of Cohort (≤117.86mg/day)	31	1	
	≥Median Dose of Cohort (≥117.86mg/day)	32	2.10 (0.76-5.84)	0.155
Route of CBMP Administration	Oils	18	1	
	Dried Flower or Both	45	3.35 (0.95-11.75)	0.059

OR - odds ratio, 95% CI - 95% confidence interval, BPI - Brief Pain Inventory, SF-MPQ-2 - Short Form McGill Pain Questionnaire-2, Pain VAS - Pain Visual Analogue Scale, BMI - body mass index, CBD – cannabidiol, THC – tetrahydrocannabinol, CBMP – cannabis-based medicinal product.
