# Supplementary material for: UK Medical Cannabis Registry: A Clinical Outcomes Analysis for Complex Regional Pain Syndrome
Source: Brain Behav. 2025 Sep 2;15(9):e70823. doi: 10.1002/brb3.70823 (PMC12405601; doi:10.1002/brb3.70823)
Supplement: Supplementary file 4 — Supporting Appendix: brb370823‐sup‐0004‐AppendixD.pdf [file BRB3-15-e70823-s001.pdf]

Appendix D: Multivariate regression for pain-specific patient-reported outcome measures. Odds ratios and 95% confidence intervals with corresponding p-values were calculated in IBM Statistical Package for Social Sciences (SPSS) version 29. P-values shown; (***=p<0.001, **=p<0.010, *=p<0.050). Green shading = p<0.050

BPI Pain Severity				
**Variable**		**n**	**OR (95% CI)**	**p-value**
**Age (years)**	18-40	29	1	
	40+	35	7.79 (1.40-43.32)	0.019*
**BMI (kg/m²)**	≤25	25	1	
	25-30	17	0.69 (0.13-3.72)	0.665
	30+	20	4.25 (0.74-24.41)	0.104
**Gender**	Female	31	1	
	Male	33	0.25 (0.06-1.08)	0.064
**Cannabis Status**	Never Used	22	1	
	Current or Ex-User	42	9.85 (0.95-101.75)	0.055
**CBD Dose**	≤Median Dose of Cohort (≤20.00mg/day)	26	1	
	≥Median Dose of Cohort (≥20.00mg/day)	38	0.72 (0.18-2.87)	0.639
**THC Dose**	≤Median Dose of Cohort (≤117.86mg/day)	32	1	
	≥Median Dose of Cohort (≥117.86mg/day)	32	1.52 (0.26-8.74)	0.641
**Route of CBMP Administration**	Oils	18	1	
	Dried Flower or Both	46	1.14 (0.10-13.03)	0.914

Pain VAS				
**Variable**		**n**	**OR (95% CI)**	**p-value**
**Age (years)**	18-40	29	1	
	40+	34	2.73 (0.89-15.65)	0.072
**BMI (kg/m²)**	≤25	25	1	
	25-30	16	0.60 (0.13-2.81)	0.520
	30+	20	3.41 (0.71-16.43)	0.127
**Gender**	Female	31	1	
	Male	32	0.65 (0.18-2.39)	0.517
**Cannabis Status**	Never Used	21	1	
	Current or Ex-User	42	28.63 (2.53-324.51)	0.007**
**CBD Dose**	≤Median Dose of Cohort (≤20.00mg/day)	26	1	
	≥Median Dose of Cohort (≥20.00mg/day)	37	0.51 (0.15-1.83)	0.303
**THC Dose**	≤Median Dose of Cohort (≤117.86mg/day)	31	1	
	≥Median Dose of Cohort (≥117.86mg/day)	32	0.56 (0.12-2.58)	0.459
**Route of CBMP Administration**	Oils	18	1	
	Dried Flower or Both	45	0.60 (0.07-5.44)	0.646

SF-MPQ-2 Overall Score				
**Variable**		**n**	**OR (95% CI)**	**p-value**
**Age (years)**	18-40	29	1	
	40+	34	1.84 (0.53-6.36)	0.334
**BMI (kg/m²)**	≤25	25	1	
	25-30	16	0.93 (0.22-3.96)	0.917
	30+	20	1.69 (0.42-6.85)	0.462
**Gender**	Female	31	1	
	Male	32	0.34 (0.10-1.21)	0.097
**Cannabis Status**	Never Used	21	1	
	Current or Ex-User	42	2.80 (0.53-14.89)	0.227
**CBD Dose**	≤Median Dose of Cohort (≤20.00mg/day)	26	1	
	≥Median Dose of Cohort (≥20.00mg/day)	37	1.43 (0.43-4.74)	0.558
**THC Dose**	≤Median Dose of Cohort (≤117.86mg/day)	31	1	
	≥Median Dose of Cohort (≥117.86mg/day)	32	0.90 (0.22-3.68)	0.879
**Route of CBMP Administration**	Oils	18	1	
	Dried Flower or Both	45	3.10 (0.42-22.79)	0.267

OR - odds ratio, 95% CI - 95% confidence interval, BPI - Brief Pain Inventory, SF-MPQ-2 - Short Form McGill Pain Questionnaire-2, Pain VAS - Pain Visual Analogue Scale, BMI - body mass index, CBD – cannabidiol, THC – tetrahydrocannabinol, CBMP – cannabis-based medicinal product.
